# Supplementary material for: The complexity of protein interactions unravelled from structural disorder
Source: PLoS Comput Biol. 2021 Jan 8;17(1):e1008546. doi: 10.1371/journal.pcbi.1008546 (PMC7846008; doi:10.1371/journal.pcbi.1008546)
Supplement: S1 Text — We discuss the details of the analysis followed to generate the Supplemental Tables and Figures. (PDF) [file pcbi.1008546.s001.pdf]

SUPPLEMENTAL INFORMATION: THE COMPLEXITY OF PROTEIN INTERACTIONS UNRAVELLED FROM STRUCTURAL DISORDER

Beatriz Seoane
Complutense University of Madrid,
Departamento de Física Teórica,
Avenida de la Complutense S/N,
28040 Madrid, Spain.
beaseobar@gmail.com

Alessandra Carbone
Sorbonne Université, CNRS, IBPS,
LCQB - UMR 7238,
4 place Jussieu, 75005 Paris, France.
alessandra.carbone@lip6.fr

A Additional examples of hierarchical organisation of interfaces

We show different examples of the hierarchical organisation of the (very) different interfaces of clusters discussed in the main text. Firstly, we reproduce Fig 1 in a larger size in S1 Fig. We also show two additional examples, corresponding to clusters 3vd1_D in S2**A** Fig and 2c8g_A in S2**B** Fig. The different interfaces available but not included in the Figures were excluded either for lack of space, in the case of S1 Fig, or because they were very hard to distinguish by eye to one of the other interfaces shown in the tree.

B On the normalisation of the B-factor

The different definitions of soft disorder given in the main text are formulated in terms of a normalised b-factor. This b-factor for each residue is obtained as

$$b = \frac{B - \mu(B)}{\sigma(B)}, \tag{1}$$

where B is the experimental B-factor of the C_α of each chain's residue, and $\mu(B)$ and $\sigma(B)$, average and standard deviation of the distribution of B values for all residues in the chain, respectively.

In the main text we argue that we use the normalised version, instead of the original one, because it avoids the dependency on the experiments' resolution, but also because it better catches the structurally disordered/structured nature of these residues. We show several examples of this in S3 Fig. Our current definition is able to distinguish better the DtO residues from the rest (see S3A and S3B Figs), the residues adjacent to missing residues (see S3C and S3D Figs) and the residues predicted as disordered by several disorder predictors (see S3E and S3F Figs).

C About the clusters composition

The list of clusters used, together with the PDB index of the structures of the chains included, the list of structures with the different interfaces in each cluster and the list of clusters with unbound forms can be downloaded online `www.lcqb.upmc.fr/disorder-interfaces/`.

A breakdown of the number of clusters considered in each group of clusters discussed in the paper is shown in Table A, where we can see the amount of clusters that contain at least one interface and the number of PDB complexes and individual chains. Also the number of clusters with or without unbound forms. In S4 Fig, we include some additional histograms concerning the number of different interfaces and unbound structures.

D List of structures of cluster 6c9c_A (Fig 4 main text)

We show in Fig 3 of the main text 13 structures for the protein chains in the cluster 6c9c_A. One of them is an unbound structure (chain A of PDB index 6c9c), and the other 12 cover are the different interfaces measured in the cluster. Their

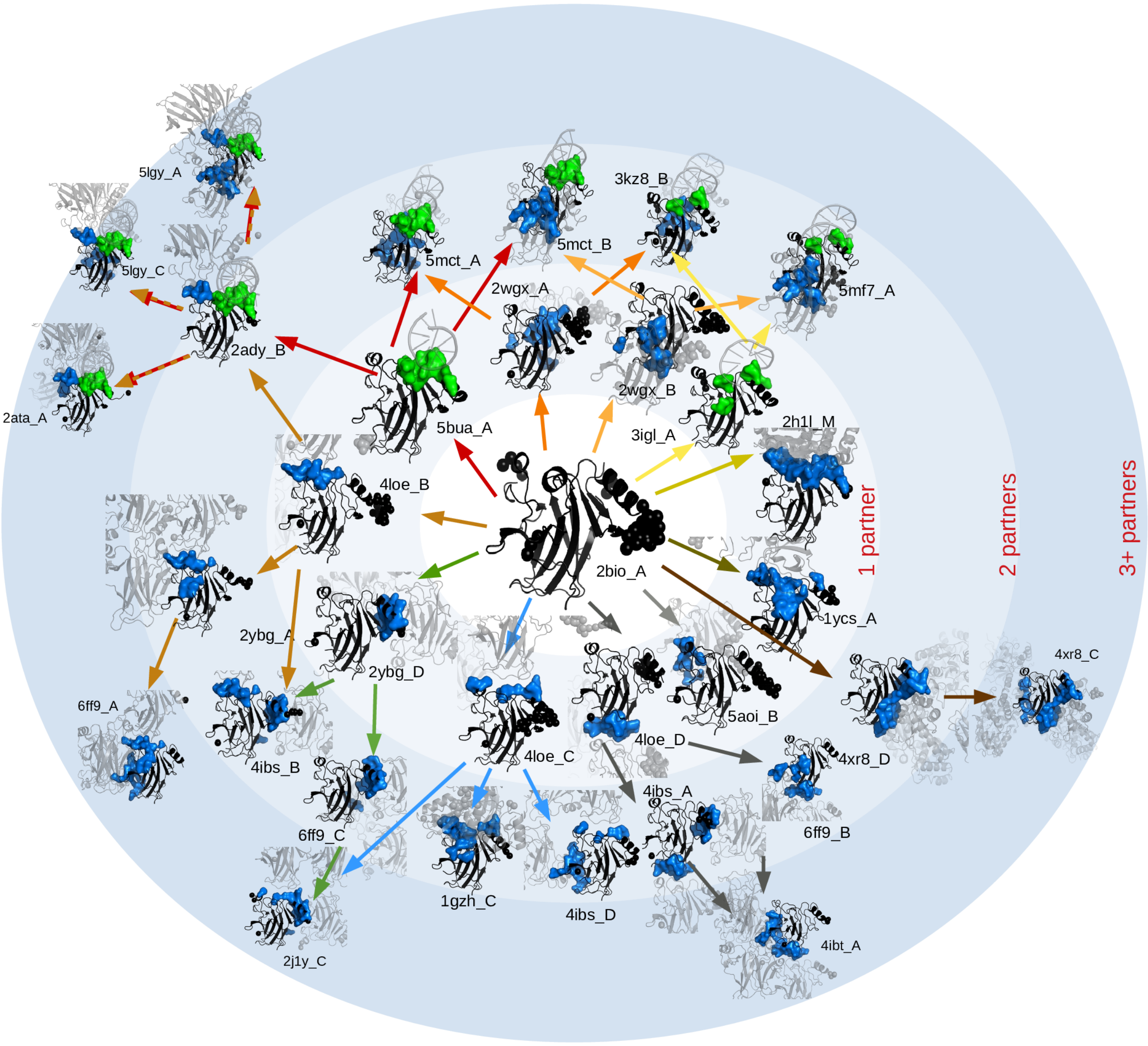

S1 Figure: **Hierarchical organisation of interfaces in the core domain of protein p53 (cluster 4ibs_A).** We reproduce in a larger format Fig 1 in the main text. Cluster 4ibs_A contains 196 structures and 42 different interfaces, among which, 27 are shown here. The rest of them were removed either for lack of space or because they were very similar to the ones shown here.

	Clusters	Clusters w/ interfaces	Complexes	Chains
All clusters	47102	37034	134337	354309
Clusters w/ unbound	15994	5926	80722	129159
Clusters w/o unbound	31108	31108	59097	225150

Table A: **Breakdown of the number of clusters** in each group of clusters discussed in the main text. We include the amount of clusters that contain at least one interface, together with the total number of PDB complexes and individual chains.

PDB indexes and chain names are 5n8c_A, 5n8c_B, 4fw3_A, 4fw3_C, 4fw4_A, 4fw4_C, 4fw5_C, 4fw7_A, 2ves_A, 3u1y_A, 3u1y_B, 4okg_A.

E Other characterisations for the cluster

Most of the figures in the main text are shown as function of the NDI. The NDI counts the number of different interfaces in the cluster with an overlap higher than the 5%. This choice was a bit arbitrary, and other elections could have been done, like reducing the threshold to 1% or plotting the curves against the cluster size. All these two options lead to noisier curves, but as we show in S6 Fig, do not change the conclusions.

F Total randomisation test of the disordered regions

In this section, we compare the statistics and prediction power of the disordered regions (DRs) and with the random guess. With this aim we proceed as follows. We read the disordered (as missing residue or high b-factor) amino acids from each chain structure in the cluster and randomise its location in the sequence. We consider two possible of such randomisations: a purely random one but not very meaningful in a biological sense, namely **(test 1)**: a random reshuffling of the DRs (by means of a random permutation of all the sites in the sequence). After this randomisation, we compute the DR as the union of all these random disordered sites in the cluster, as done in the main text, and compare its properties with those of the experimental IRs and its power to predict IRs. In S7**A** Fig, we show the mean relative size of these DRs for the two sets. Clearly, the behaviour of these fake disordered regions with the number of interfaces in the cluster, whose sizes cover essentially the whole chain above 10 interfaces, differs strongly with the data shown for the union of the interface regions (or the real disordered regions).

We compare the data shown in Fig 7 in the main text, concerning the number of connected interfaces or disordered regions of our clusters (normalised by the sequence size), with the number of clusters that one would measure if the DRs were random. We consider two distinct tests: (**test 3**), a random permutation of the DR sites in the sequence, and (**test 4**), a reshuffling of the DRs keeping together all sequential disordered sites, keeping in both cases the total number of disordered sites fixed. We show in S7**B** Fig the averaged number of this number of connected regions among all the clusters with the same number of different interfaces. Again, both tests give significantly different curves than the real ones, as long as the NDI is not too high (where the disordered regions superimpose forming one or two very large clusters).

G Additional Figures

A

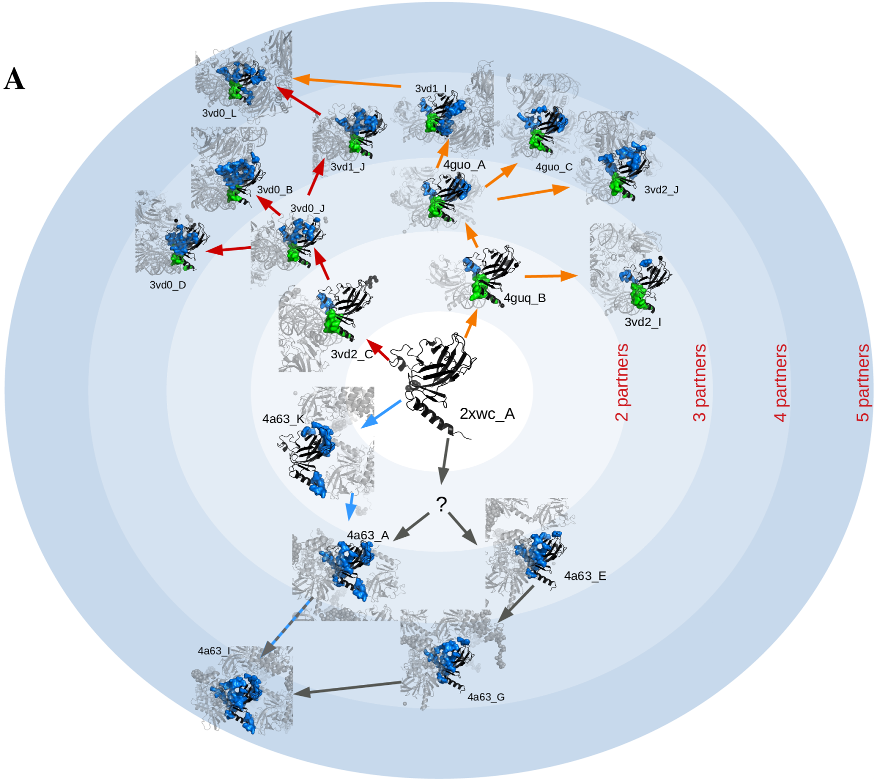

B

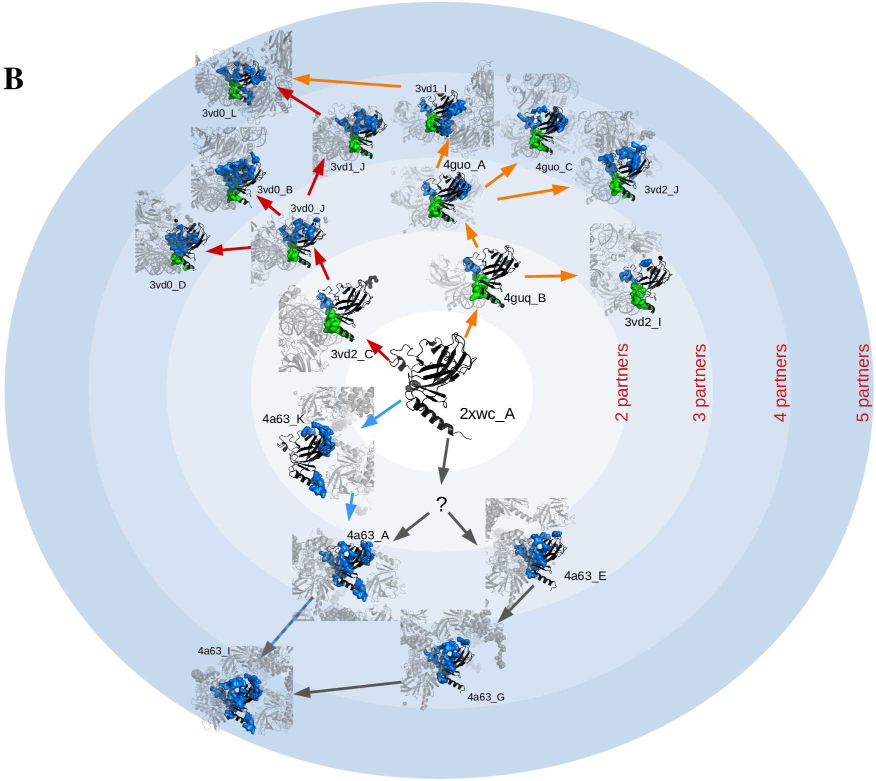

S2 Figure: **Other hierarchical interface organisation**. **A** for the p73 DNA binding domain (cluster 3vd1_D). Cluster 3vd1_D has 45 structures and 24 different interfaces (the rest of interfaces not shown are hard to distinguish from those displayed). **B** for the C3 exoenzym (cluster 2c8g_A). Cluster 2c8g_A has 47 structures and 10 different interfaces (all shown here).

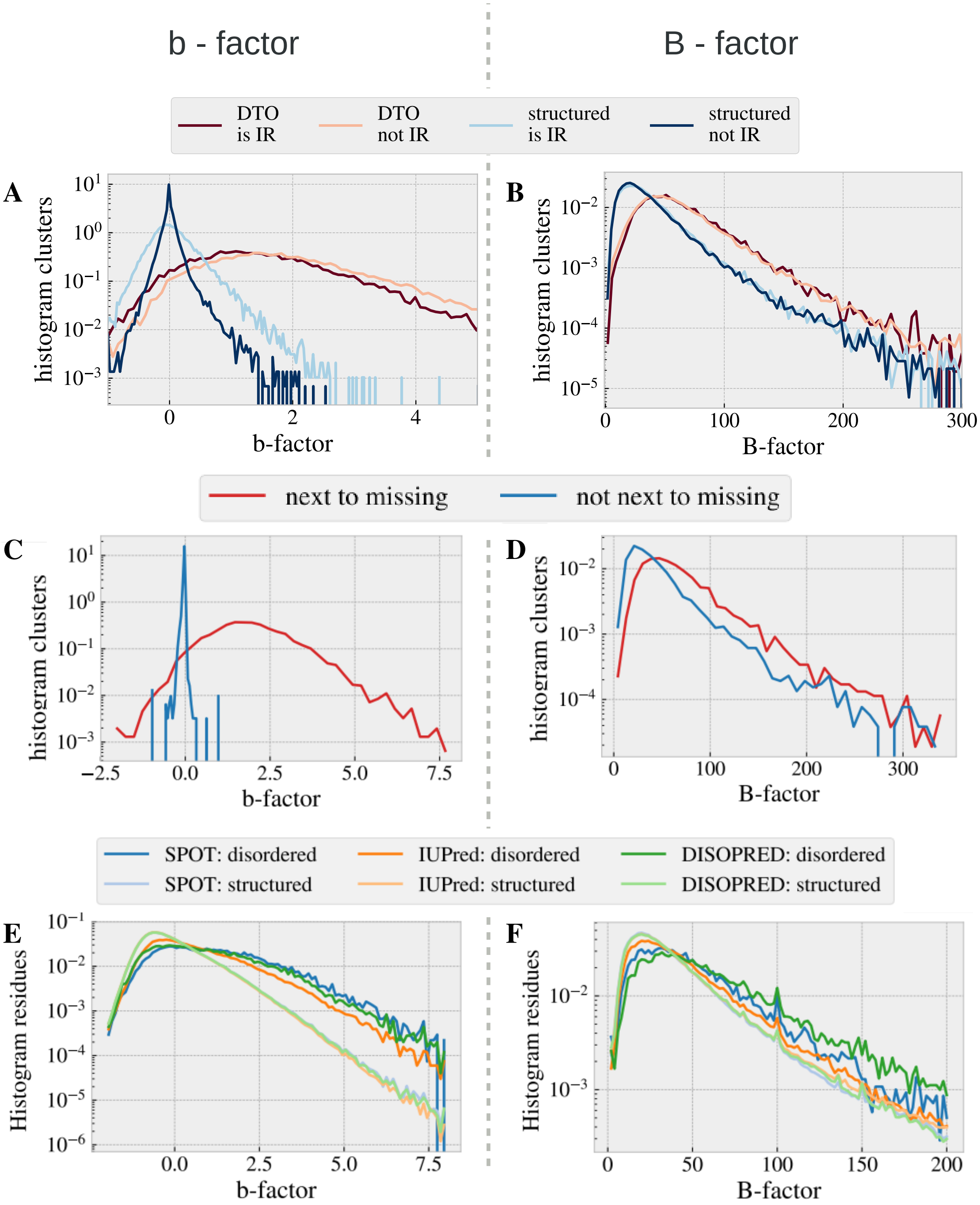

S3 Figure: **On the normalisation of the B-factor.** We show two columns of figures, the left column is computed using with the normalised b-factor, and the right column using the experimental B-factor. In **A and B**, we show a histogram of the mean value (in each of the set of clusters) of the b-factor of the residues that belong either belong to the DtO or not, and grouped separately if each residue was or not part of the interface for each of the structures of the cluster. Fig **A** shows the same data that Fig 6 in the main text, but in logarithmic scale. In **C and D**, we show an histogram of the mean value (for each cluster) of the b-factor of the residues that were (or were not) just next to a missing residue in the sequence. In **E and F**, we show the histogram of the b-factor of each of the residues o the cluster representative structure predicted (or not predicted) as disordered by the three predictors considered in the main text.

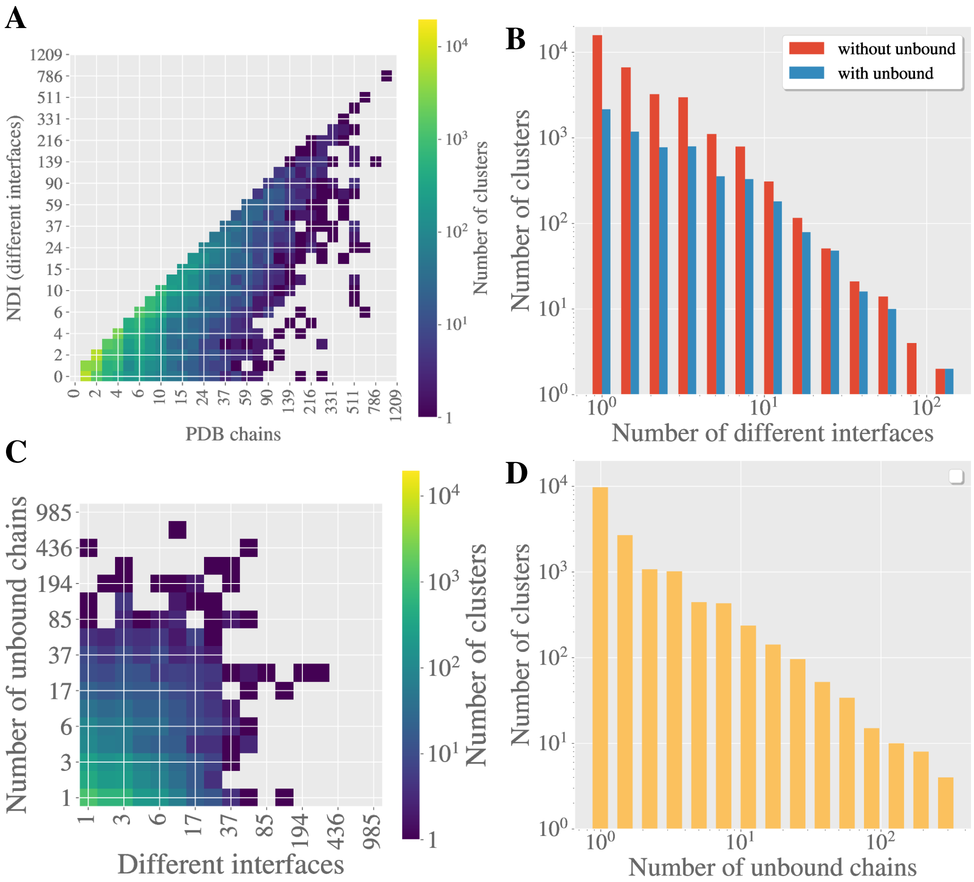

S4 Figure: **Histograms of the cluster composition.** **A** Bi-dimensional histogram of the number of clusters with a given number of different interfaces and a given size. **B** Number of clusters as function of the number of different interfaces for the groups of clusters that either contain any unbound chain or they do not. **C** Bi-dimensional histogram of the number of unbound chains in the cluster as function of the number of different interfaces. **D** Number of clusters as function of the number of unbound chains contained in each cluster (only clusters with unbound structures considered).

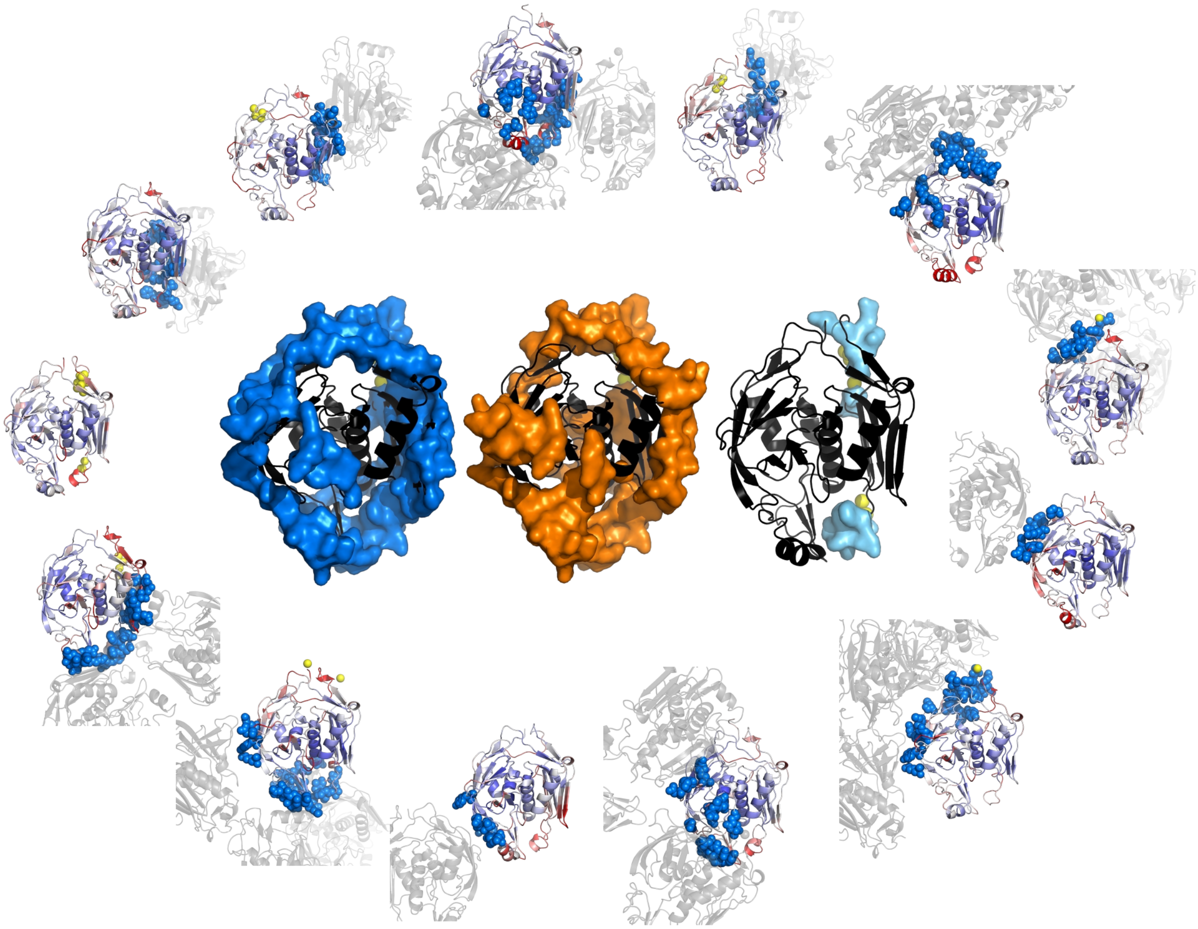

S5 Figure: **Cluster representation.** We reproduce the same cluster of Fig 4, but this time showing the partners of each protein chain (in a grey shadow), the binding sites as blue spheres and the b-factor of the chain through a colour code (being deep red very high b-factor and deep blue, very low b-factor). In the centre, we show again the union of all the interface (blue), soft disorder (orange) and disorder-to-order (light blue) regions.

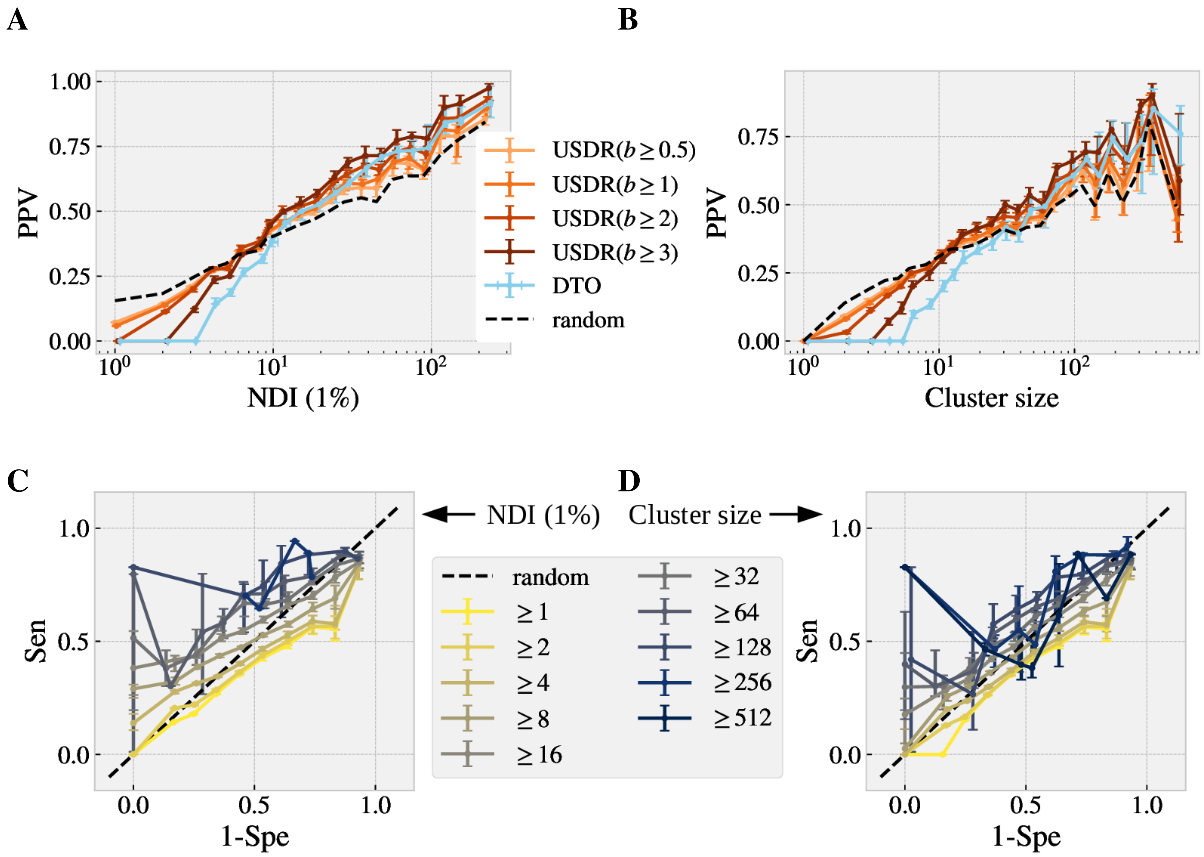

S6 Figure: **Other characterisation of the cluster content.** We repeat some of the curves of the main text, but this time showing the results as a function of the the number of different interfaces up to the 1% of the sequence (**A** and **C**), or the size of the cluster (**B** and **D**). In **A** and **B** we show the figures analogous to Fig 8B of the main text, and in **C** and **D**, the figures analogous to Fig 9B

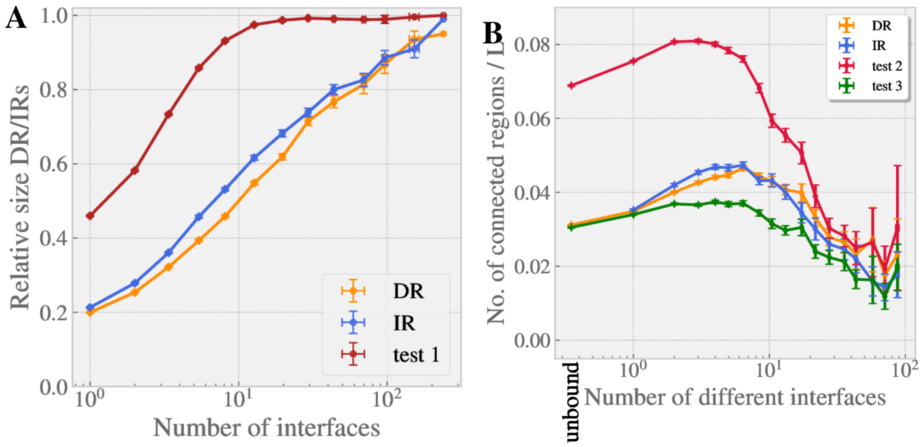

S7 Figure: **Randomisation tests.** In **A**, we compare the averaged relative size of union of disordered (orange) and interface (blue) regions (shown in Fig 7A in the main text) with respect to the sequence length as function of the cluster size, with the averaged relative size of the union of fake disordered regions obtained after reshuffling the experimental disordered regions (test 1, red). The randomised disordered regions follow a rather different behaviour with the number of interfaces than the union of experimental interface regions. In **B**, we compare the averaged number of connected disordered regions (DR, orange) and interface regions (IR, blue), normalised by the sequence length, as function of the number of different interfaces in the cluster with the numbers we would obtain if the same number of disordered sites where randomly distributed. We have considered two distinct randomisation tests: a random permutation of the disordered sites in the sequence (test 2) and a reshuffling of the disordered regions but keeping consecutive disordered sites together (test 3). Both tests lead to different curves than the real ones, with the exception of the very big clusters, where the regions superimpose forming a very large cluster.

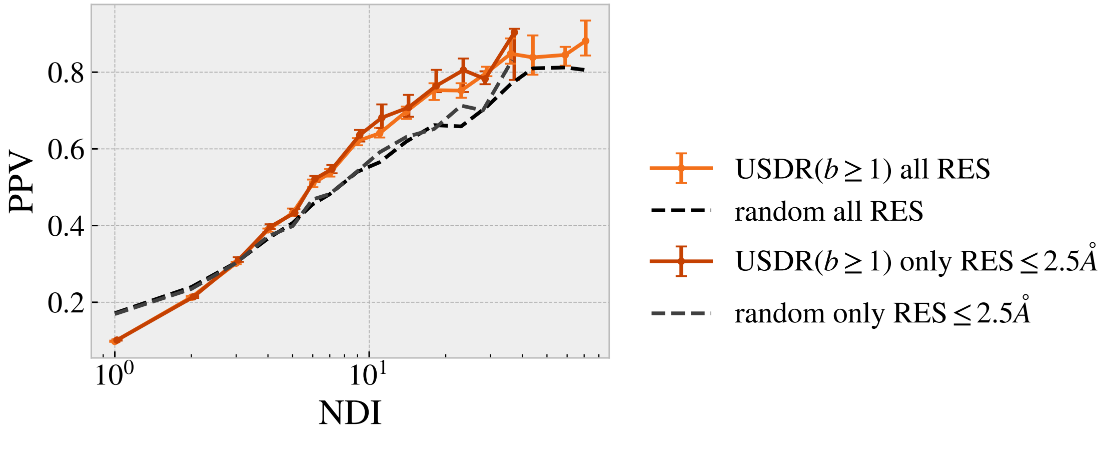

S8 Figure: **Clusters of high resolution structures.** We compare the PPV (medians by bin) for the USDR($b > 1$) shown in Fig 8B of the main-text (light orange, computed using all the structures of the PDB), with the values we obtain if clusters are just composed of structures with resolution below 2.5Å(dark orange). In dash lines, we show the median expected PPV for a trivial correlation using all structures (black) and structures with high resolution (grey), displaying essentially the same curves. As show, we observe no significant change in the correlation between soft disorder and interfaces with the resolution, despite the fact that curves are now noisier in the high resolution case, because there are less clusters with a high number of structures than in the case studied in the paper.

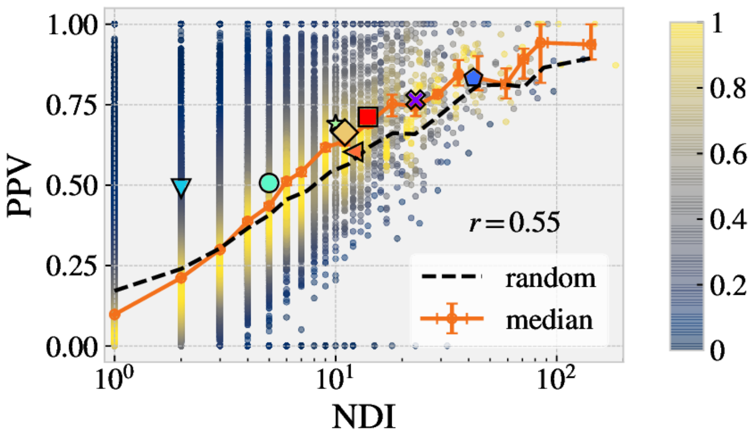

S9 Figure: **Metrics excluding the once missing residues.** We repeat Fig 8A but this time excluding from the analysis the residues that are reported as missing at least in one structure of the cluster. The results are indistinguishable from the ones containing DtO residues, thus excluding the possibility that the signal reported is trivially introduced by DtO residues forming interfaces.

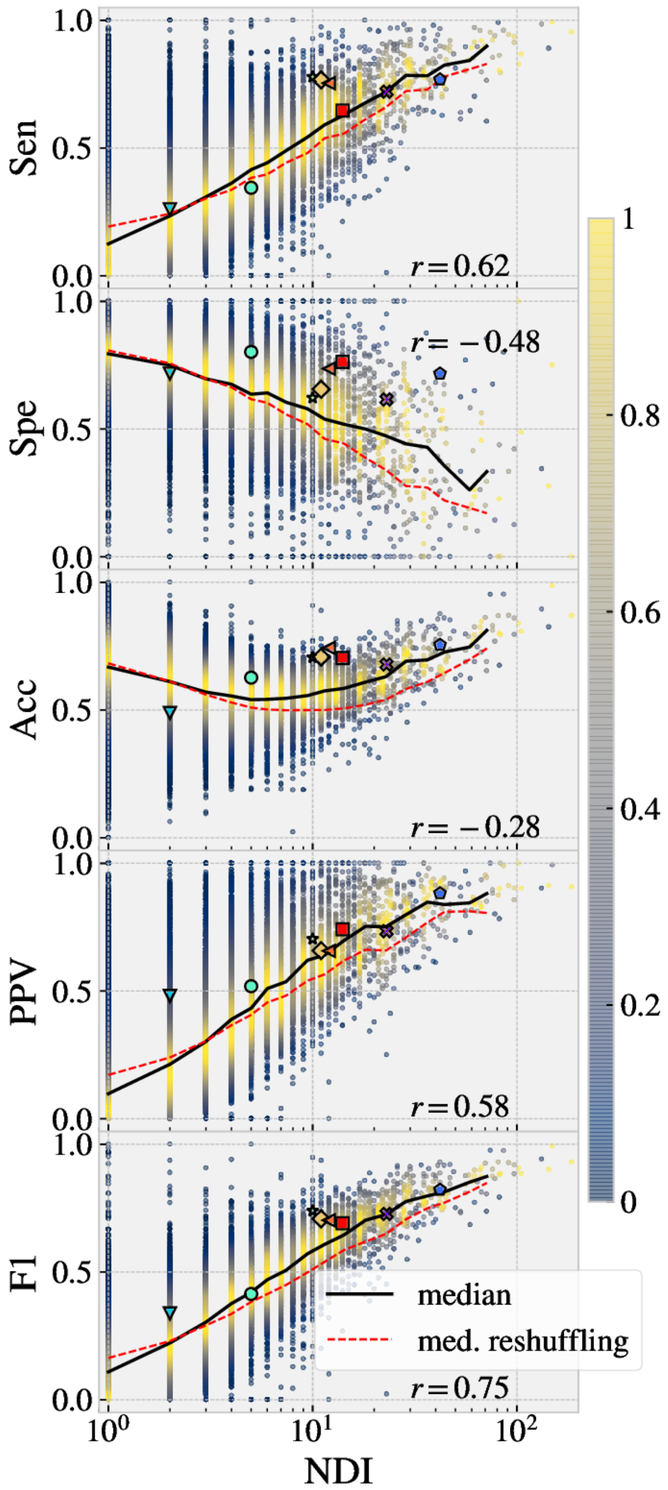

S10 Figure: **Metrics of the goodness of the match between the USDR and the UIR.** We reproduce the analogous curve to Fig 8A for other possible metrics, including the Sensibility (Sen), the Specificity (Spe), the Accuracy (Acc), the positive prediction value (PPV) and the F1 metrics, which is given by the harmonic mean between the Sensitivity and the PPV. The colour dots correspond to the structures shown in Fig 8C.

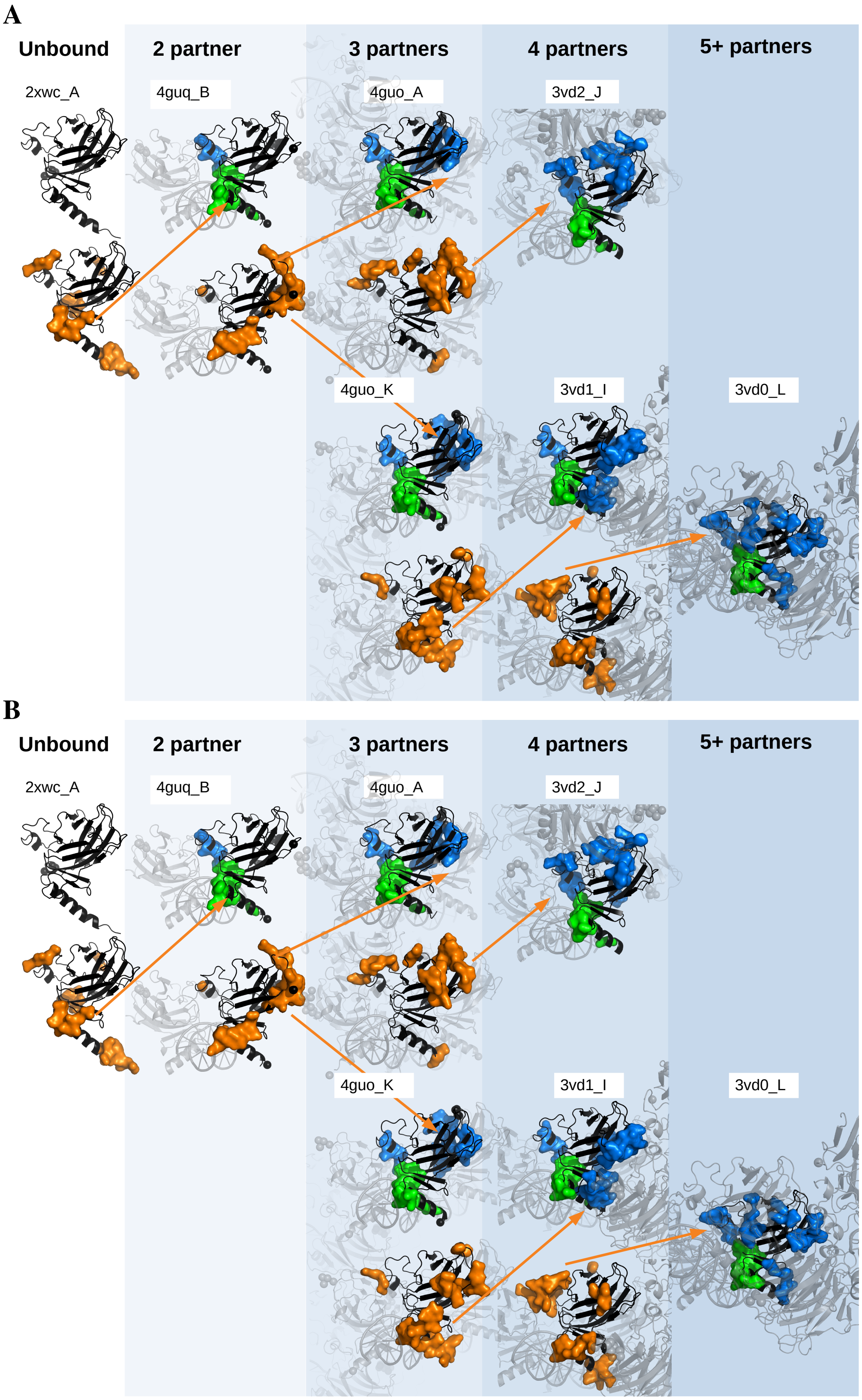

S11 Figure: **Progressive change of DRs upon binding**. **A** We show the change of DRs and IRs in the p73 DNA binding domain (cluster 3vd1_D) along the orange tree branch of S2**A** Fig. In **B**, the change in the C3 exoenzym (cluster 2c8g_A) along the red tree branch of S2**B** Fig.

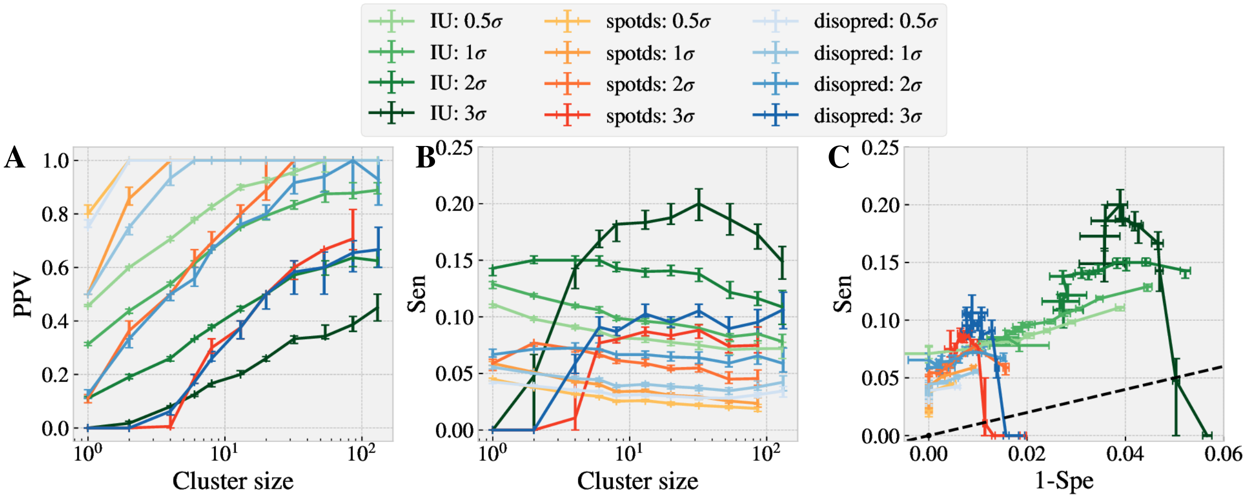

S12 Figure: **Disorder predictors and soft disorder.** We compare the predictions from the disorder predictors discussed in the main text, once removed the forever missing residues of the cluster, with our measures of the USDR. In **A** we show the PPV of the disorder predictions with respect to the different definitions of soft disorder, as function of the NDI. In **B**, we show instead the Sensibility. In **C**, we show the Sensibility versus the Specificity for the different NDI bins.

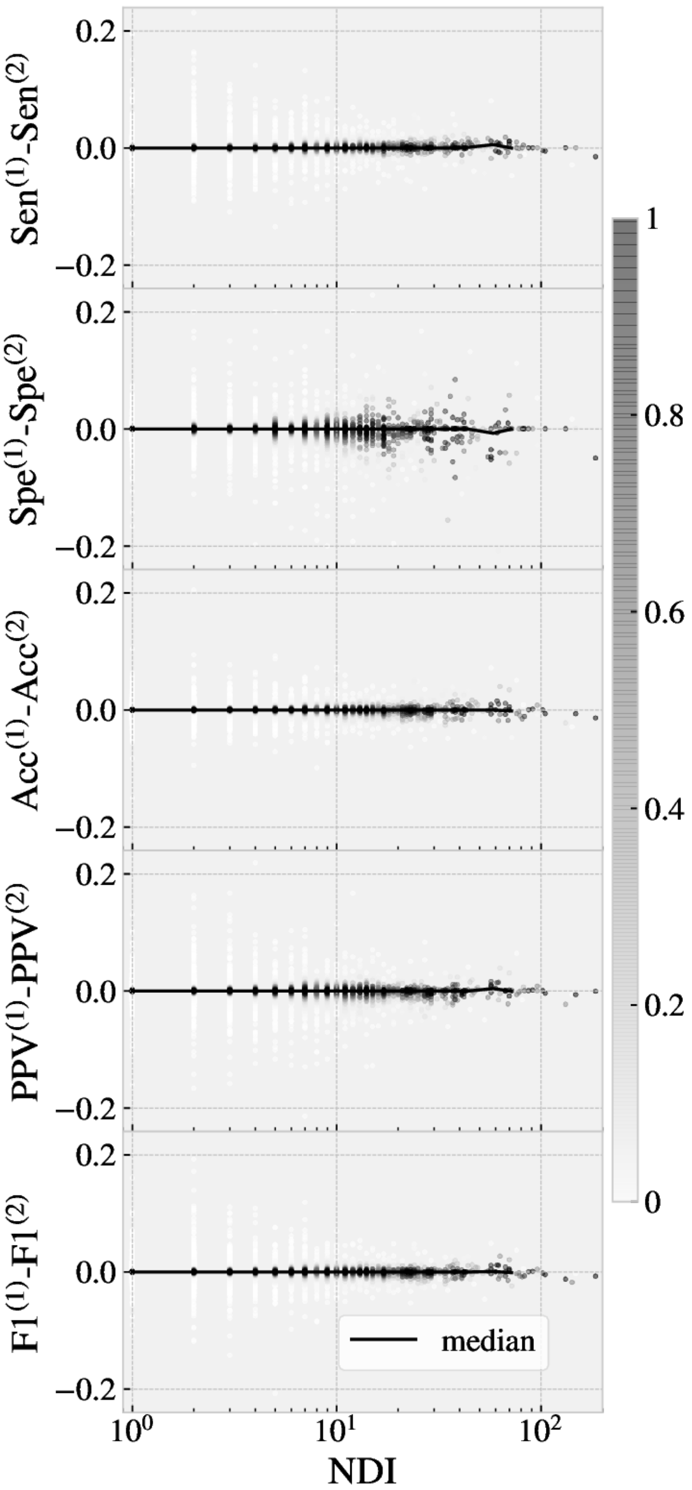

S13 Figure: **Random choice of the representative chain of the cluster**. We compare the metrics quantifying the agreement of USDR and the UIR shown up to this moment, or if the representative is chosen randomly (instead of by the clustering algorithm). Each cluster is shown as a dot, and the shade of colour indicate the density of points for each bin. We see that the choice of the representative has no systematic effect in the results.
