# Supplementary figures and images for: The complexity of protein interactions unravelled from structural disorder
Source: PLoS Comput Biol. 2021 Jan 8;17(1):e1008546. doi: 10.1371/journal.pcbi.1008546 (PMC7846008; doi:10.1371/journal.pcbi.1008546)

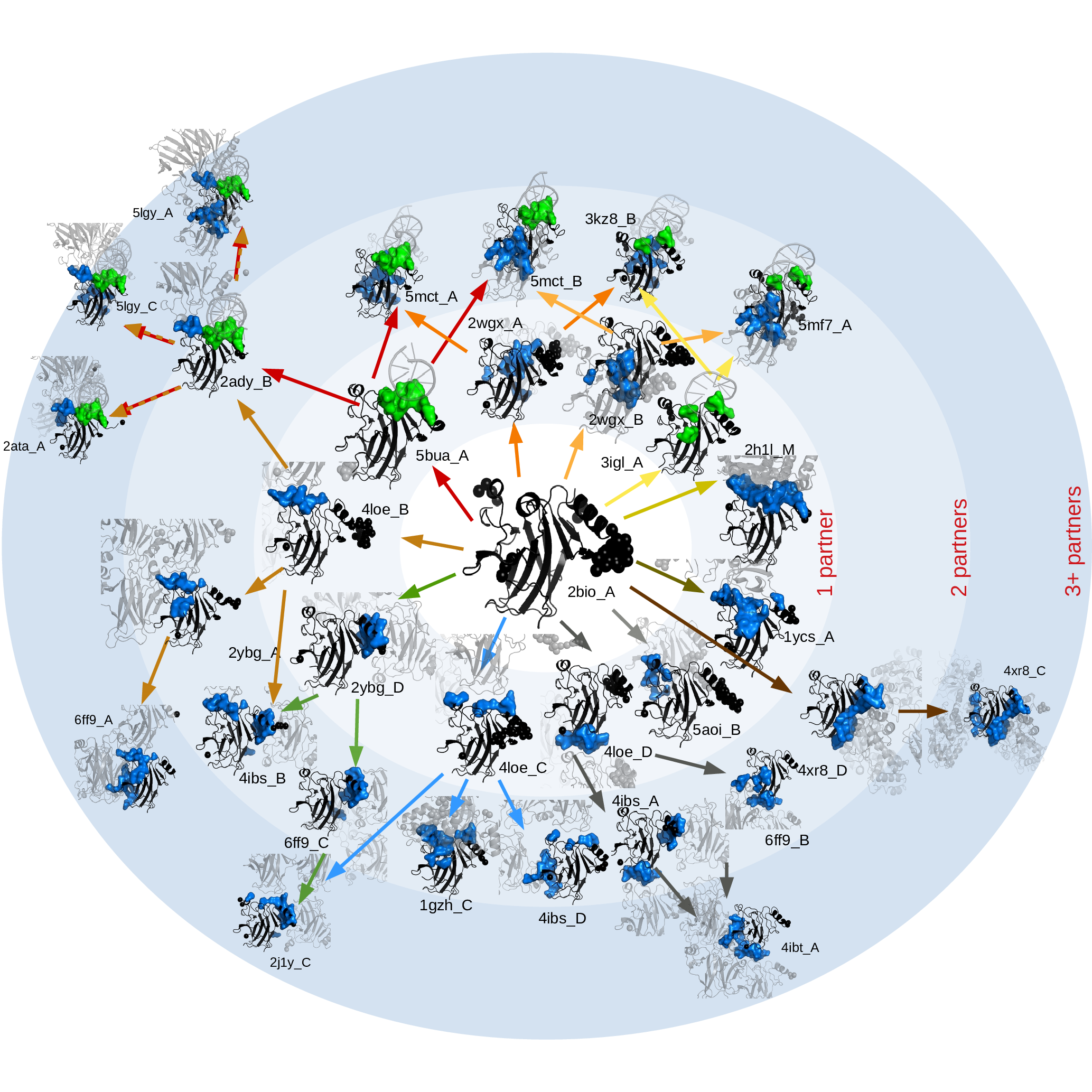

Supplement: S1 Fig — We reproduce in a larger format Fig 2 in the main text. Cluster 4ibs_A contains 196 structures and 42 different interfaces, among which, 27 are shown here. The rest of them were removed either for lack of space or because they were very similar to the ones shown here. Extra details are given in the S1 Text. (PNG) [file pcbi.1008546.s002.png]

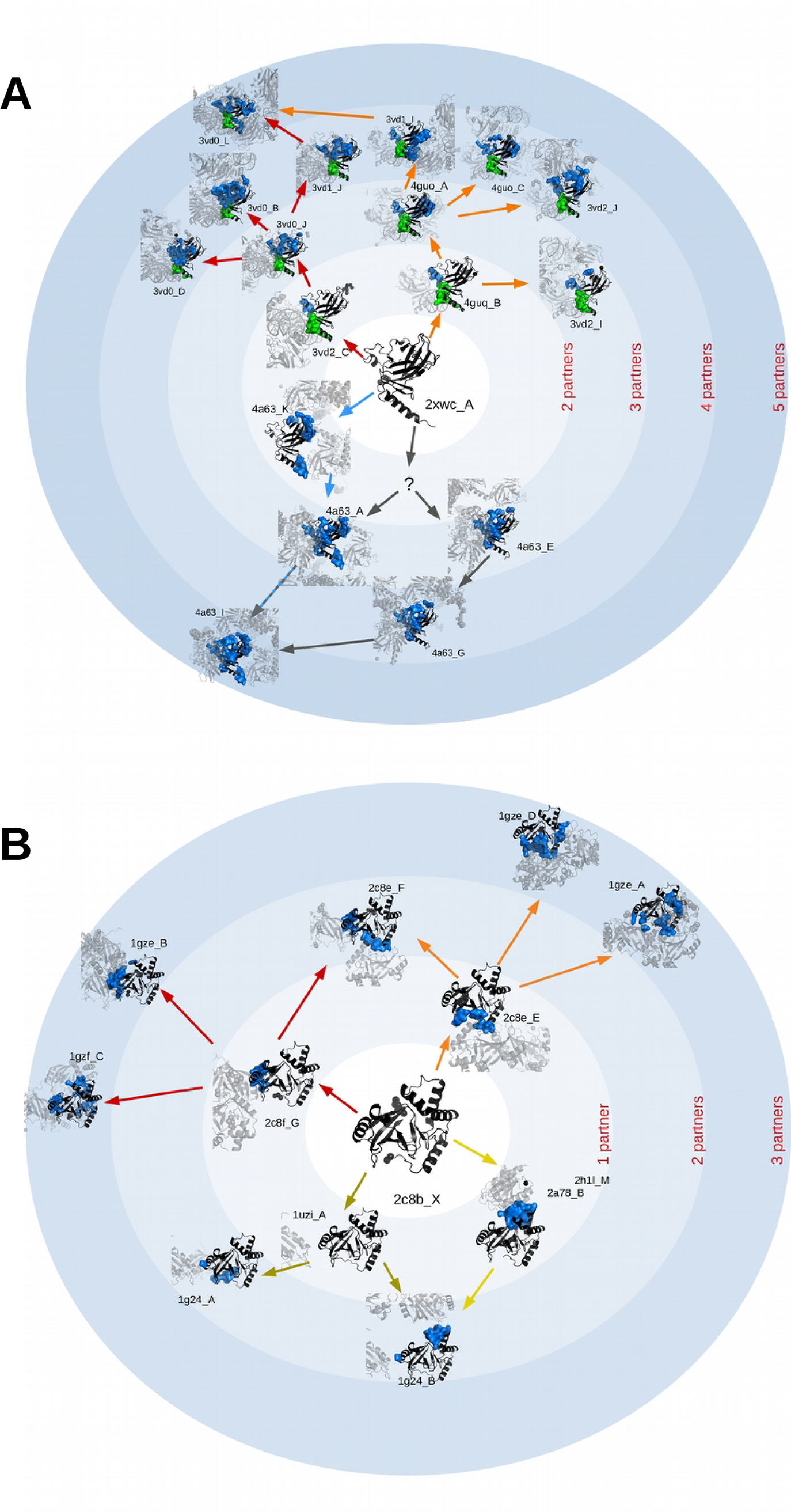

Supplement: S2 Fig — A for the p73 DNA binding domain (cluster 3vd1_D). Cluster 3vd1_D has 45 structures and 24 different interfaces (the rest of interfaces not shown are hard to distinguish from those displayed). B for the C3 exoenzym (cluster 2c8g_A). Cluster 2c8g_A has 47 structures and 10 different interfaces (all shown here). More details are given in the S1 Text. (TIF) [file pcbi.1008546.s003.tif]

b - factor

B - factor

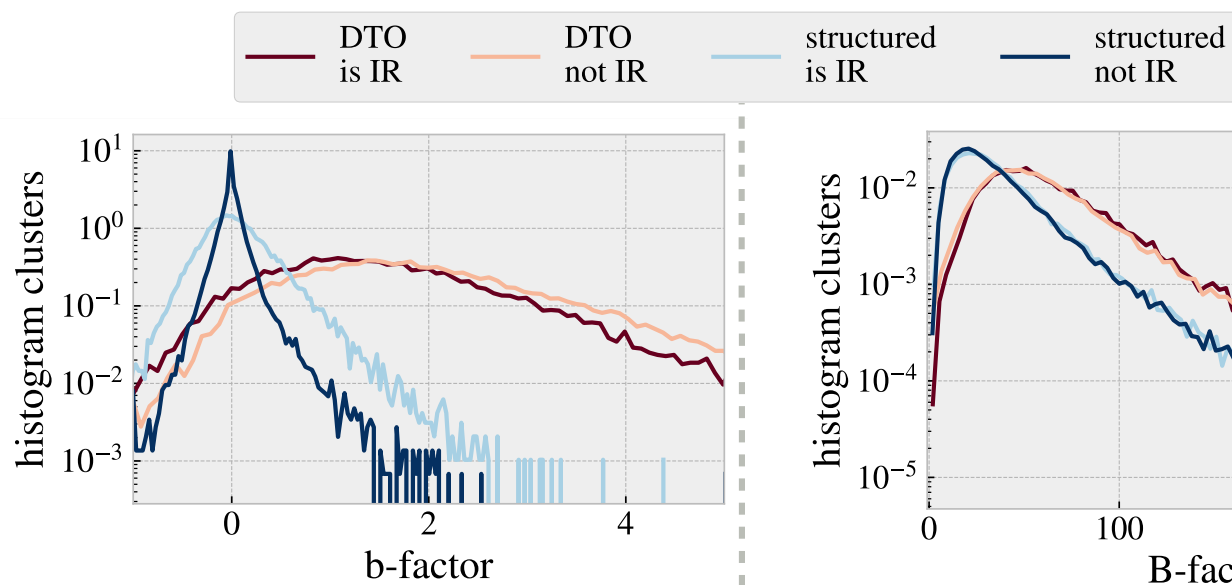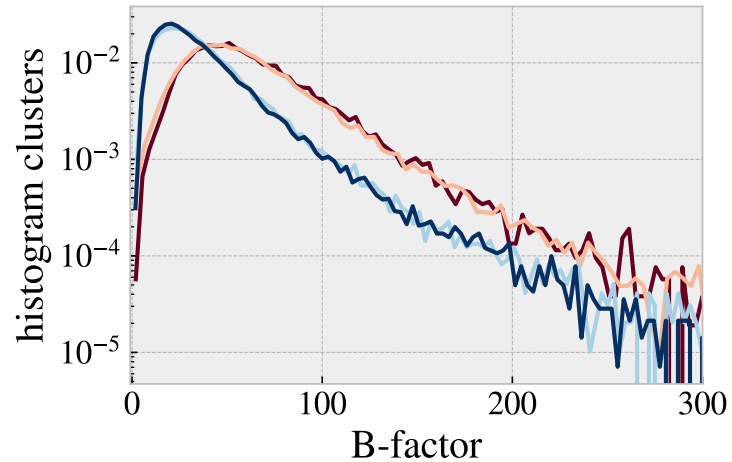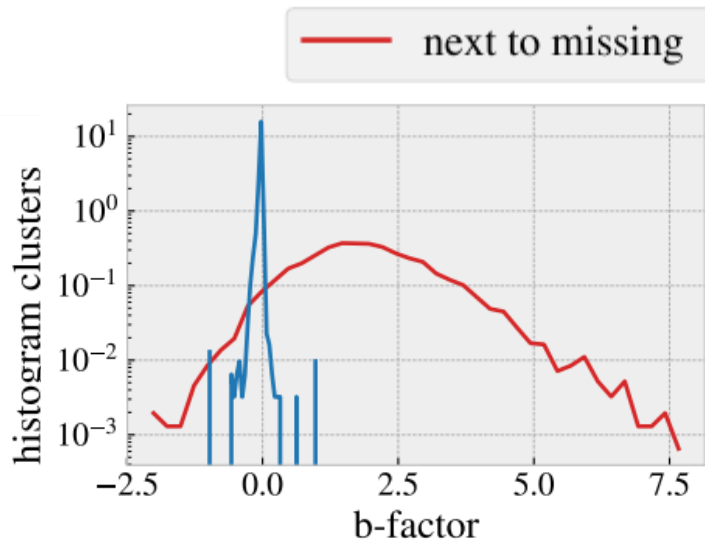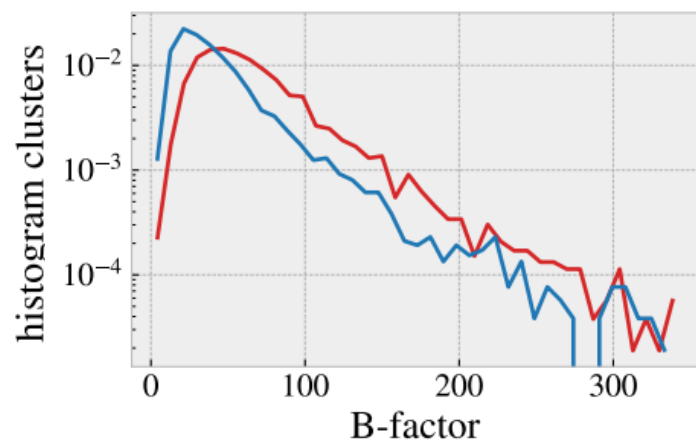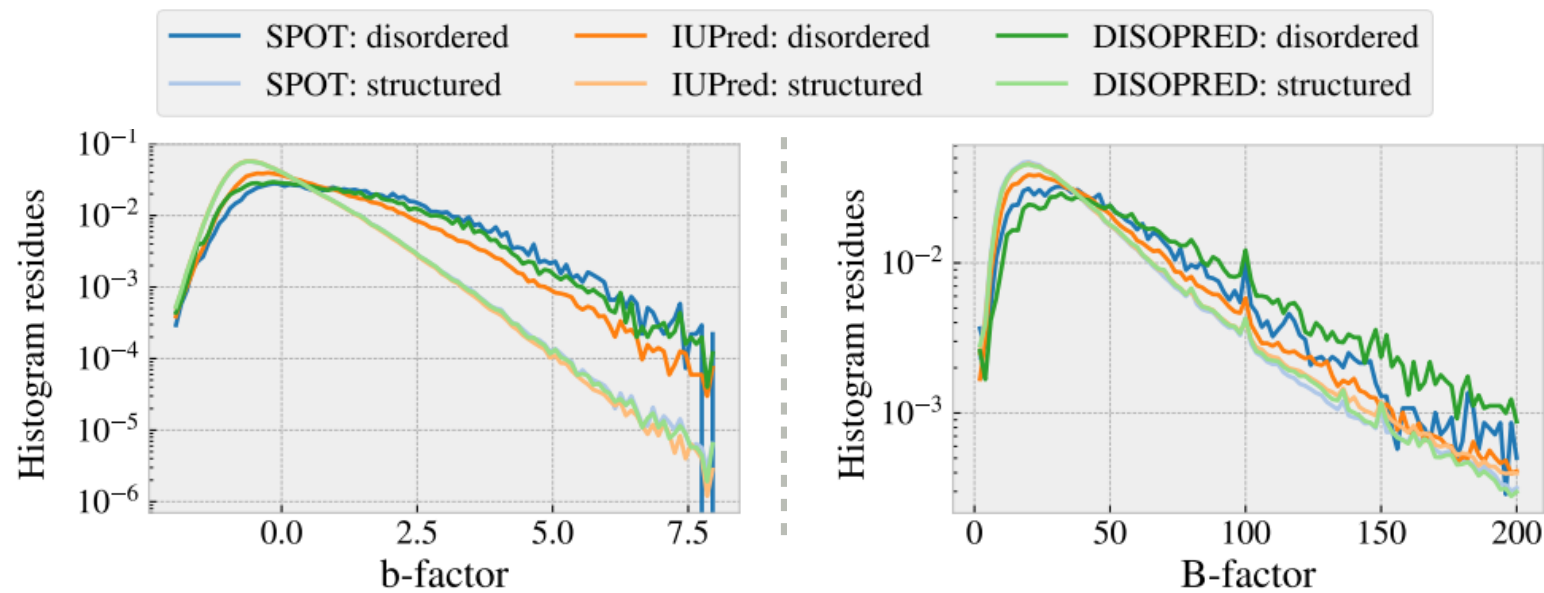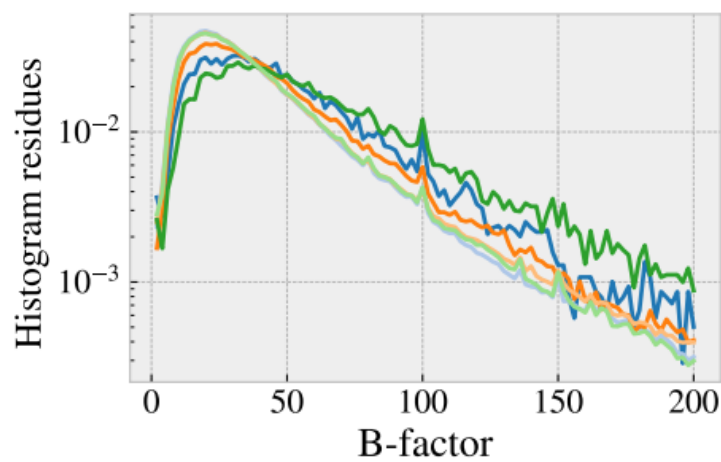

Supplement: S3 Fig — We show two columns of figures, the left column is computed using with the normalised b-factor, and the right column using the experimental B-factor. In A and B, we show a histogram of the mean value (in each of the set of clusters) of the b-factor of the residues that belong either belong to the DtO or not, and grouped separately if each residue was or not part of the interface for each of the structures of the cluster. Fig A shows the same data that Fig 6 in the main-text, but in logarithmic scale. In C and D, we show an histogram of the mean value (for each cluster) of the b-factor of the residues that were (or were not) just next to a missing residue in the sequence. In E and F, we show the histogram of the b-factor of each of the residues o the cluster representative structure predicted (or not predicted) as disordered by the three predictors considered in the main-text. More details are given in the S1 Text. (PDF) [file pcbi.1008546.s004.pdf]

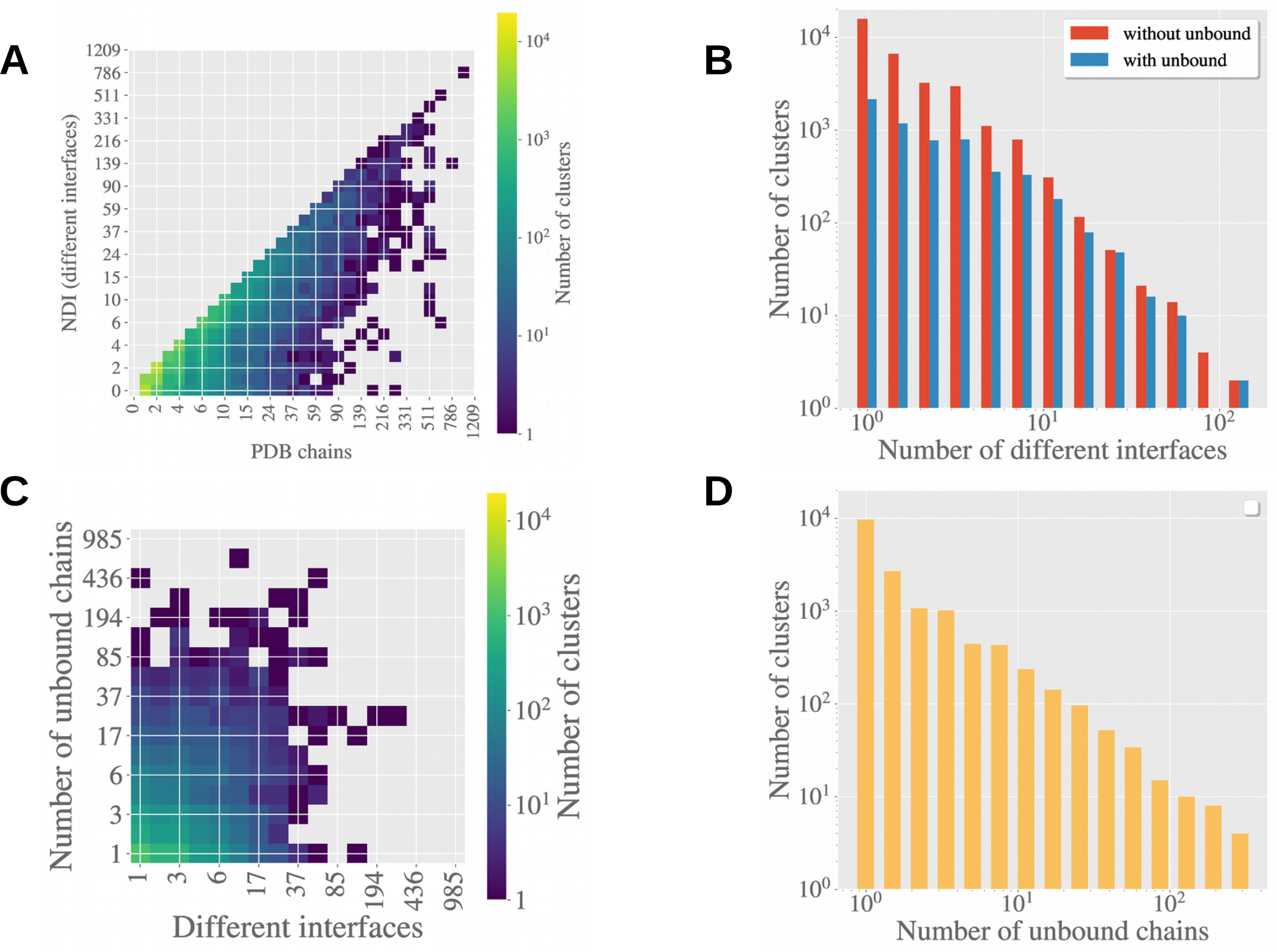

Supplement: S4 Fig — We reproduce the same cluster of Fig 4, but this time showing the partners of each protein chain (in a grey shadow), the binding sites as blue spheres and the b-factor of the chain through a colour code (being deep red very high b-factor and deep blue, very low b-factor). In the centre, we show again the union of all the interface (blue), soft disorder (orange) and disorder-to-order (light blue) regions. More details are given in the S1 Text. (TIF) [file pcbi.1008546.s005.tif]

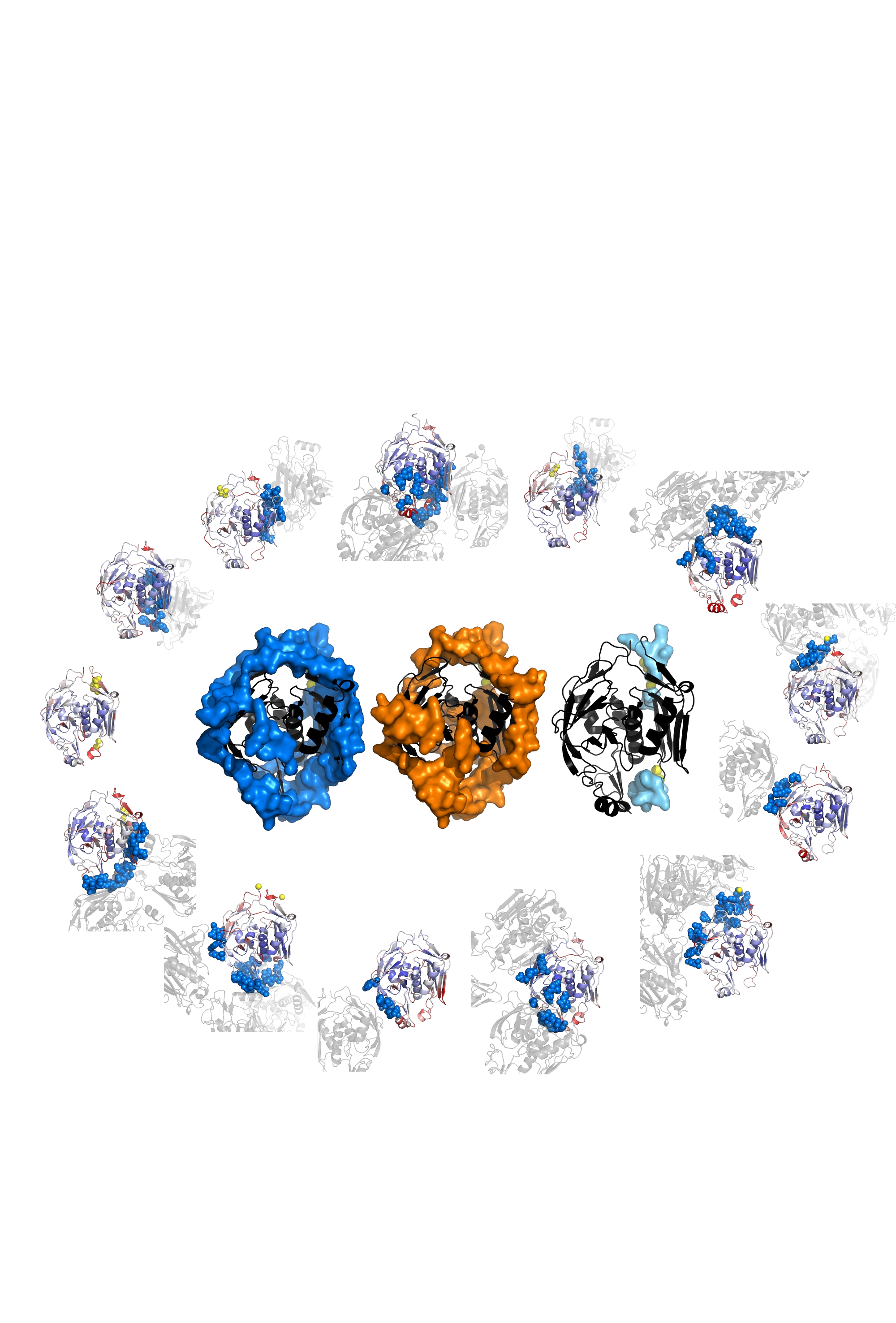

Supplement: S5 Fig — A Bi-dimensional histogram of the number of clusters with a given number of different interfaces and a given size. B Number of clusters as function of the number of different interfaces for the groups of clusters that either contain any unbound chain or they do not. C Bi-dimensional histogram of the number of unbound chains in the cluster as function of the number of different interfaces. D Number of clusters as function of the number of unbound chains contained in each cluster (only clusters with unbound structures considered). More details are given in the S1 Text. (PNG) [file pcbi.1008546.s006.png]

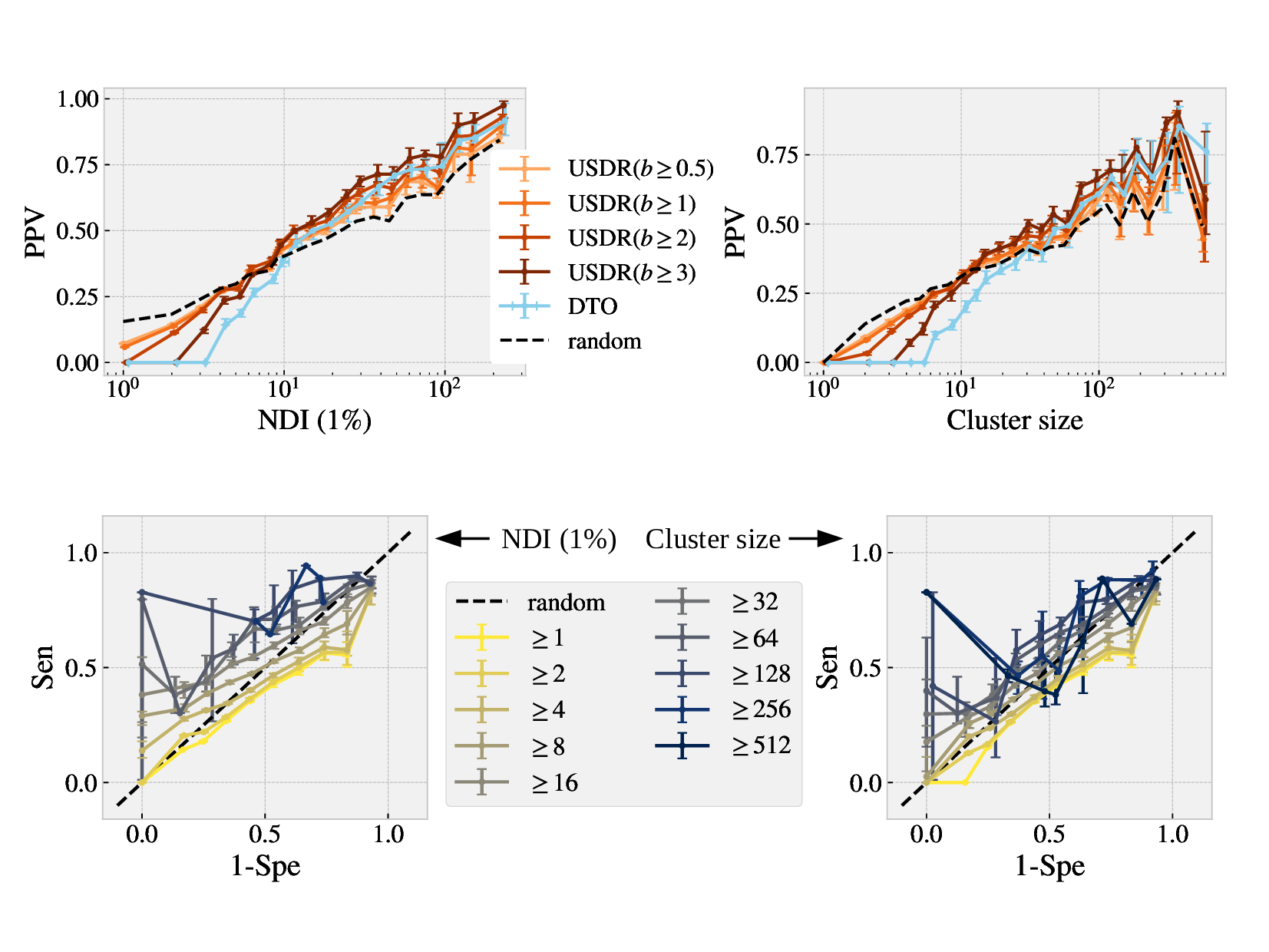

Supplement: S6 Fig — We repeat some of the curves of the main-text, but this time showing the results of as function of the the number of different interfaces up to the 1% of the sequence (A and C), or the size of the cluster (B and D). In A and B we show the figures analogous to Fig 8B of the main text, and in C and D, the figures analogous to Fig 9B. More details are given in the S1 Text. (PNG) [file pcbi.1008546.s007.png]

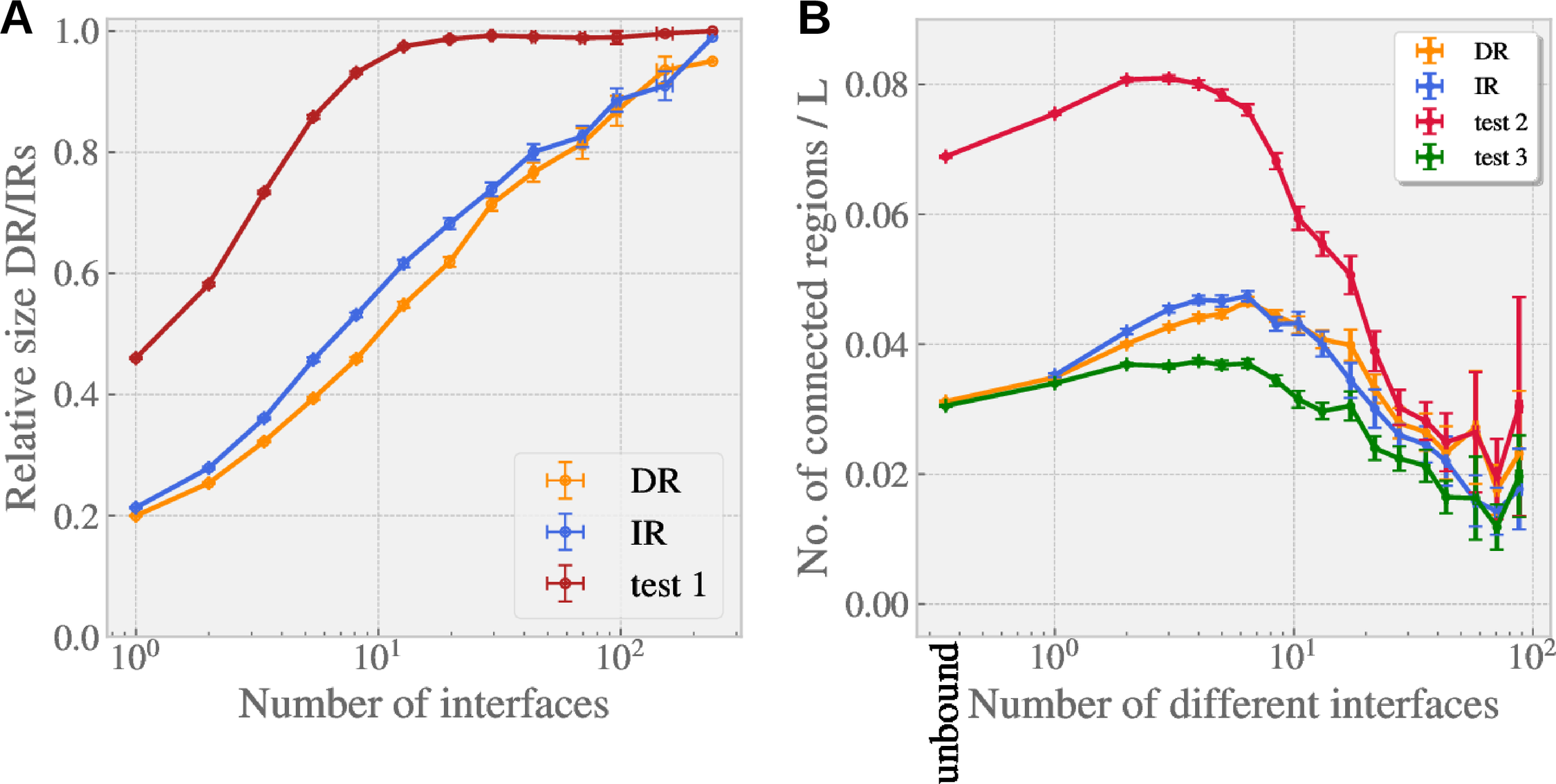

Supplement: S7 Fig — In A, we compare the averaged relative size of union of disordered (orange) and interface (blue) regions (shown in Fig 7A in the main-text) with respect to the sequence length as function of the cluster size, with the averaged relative size of the union of fake disordered regions obtained after reshuffling the experimental disordered regions (test 1, red). The randomised disordered regions follow a rather different behaviour with the number of interfaces than the union of experimental interface regions. In B, we compare the averaged number of connected disordered regions (DR, orange) and interface regions (IR, blue), normalised by the sequence length, as function of the number of different interfaces in the cluster with the numbers we would obtain if the same number of disordered sites where randomly distributed. We have considered two distinct randomisation tests: a random permutation of the disordered sites in the sequence (test 2) and a reshuffling of the disordered regions but keeping consecutive disordered sites together (test 3). Both tests lead to different curves than the real ones, with the exception of the very big clusters, where the regions superimpose forming a very large cluster. More details are given in the S1 Text. (TIF) [file pcbi.1008546.s008.tif]

PPV

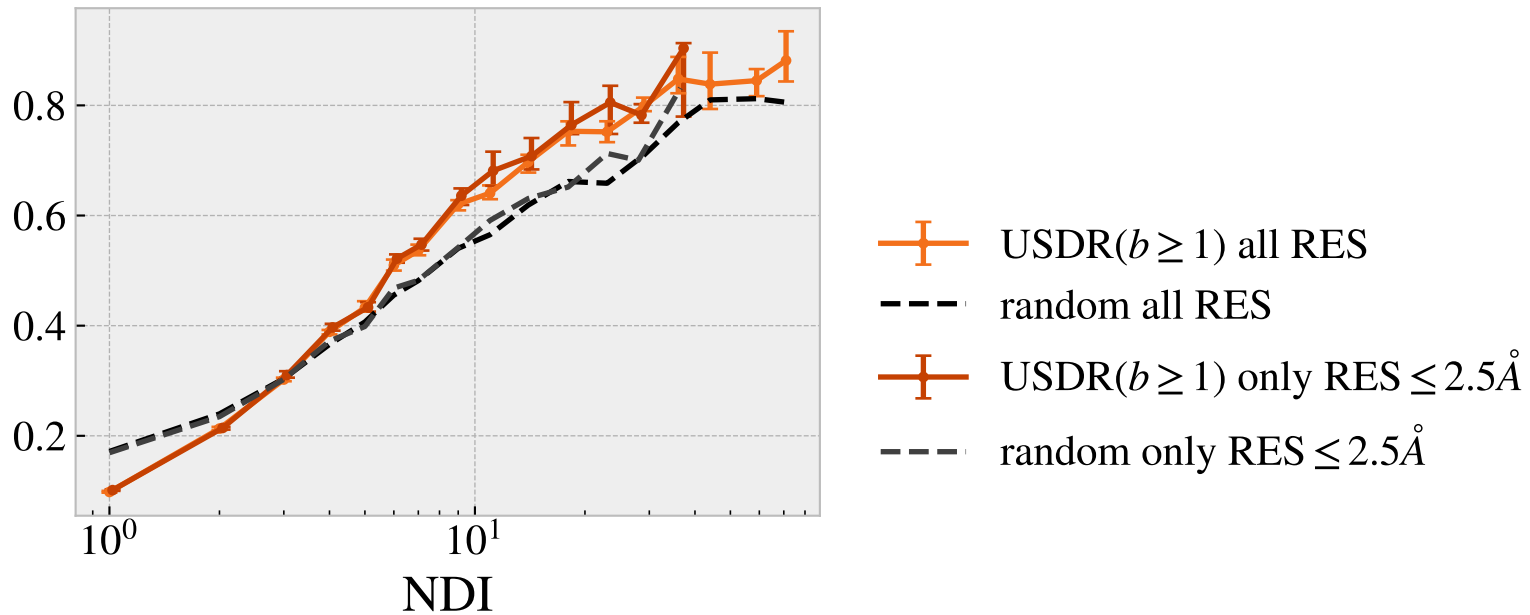

Supplement: S8 Fig — We compare the PPV (medians by bin) for the USDR(b > 1) shown in Fig 8B of the main-text (light orange, computed using all the structures of the PDB), with the values we obtain if clusters are just composed of structures with resolution below 2.5Å(dark orange). In dash lines, we show the median expected PPV for a trivial correlation using all structures (black) and structures with high resolution (grey), displaying essentially the same curves. As show, we observe no significant change in the correlation between soft disorder and interfaces with the resolution, despite the fact that curves are now noisier in the high resolution case, because there are less clusters with a high number of structures than in the case studied in the paper. (PDF) [file pcbi.1008546.s009.pdf]

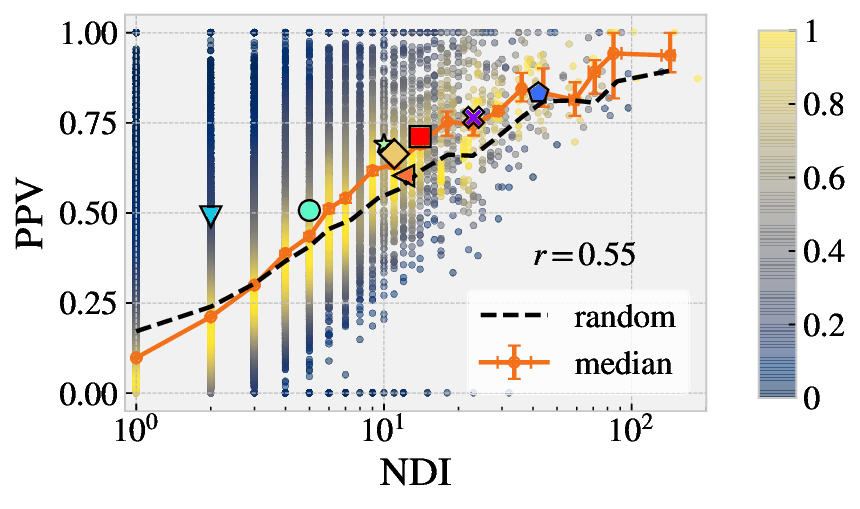

Supplement: S9 Fig — We reproduce the analogous curve to Fig 8A for other possible metrics, including the Sensibility (Sen), the Specificity (Spe), the Accuracy (Acc), the positive prediction value (PPV) and the F1 metrics, which is given by the harmonic mean between the Sensitivity and the PPV. The colour dots correspond to the structures shown in Fig 8C. (PNG) [file pcbi.1008546.s010.png]

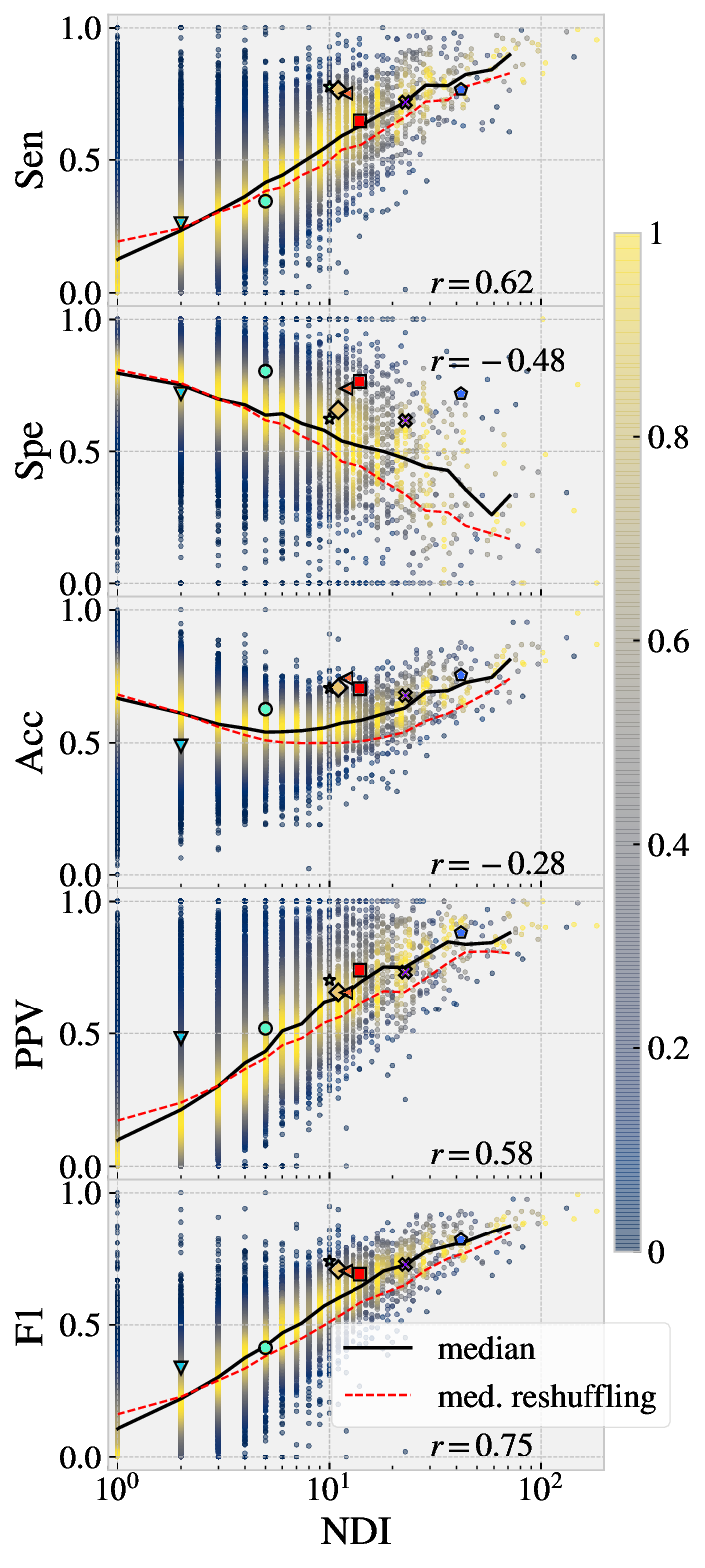

Supplement: S10 Fig — We repeat Fig 8A but this time excluding from the analysis the residues that are reported as missing at least in one structure of the cluster. The results are indistinguishable from the ones containing DtO residues, thus excluding the possibility that the signal reported is trivially introduced by DtO residues forming interfaces. (PNG) [file pcbi.1008546.s011.png]

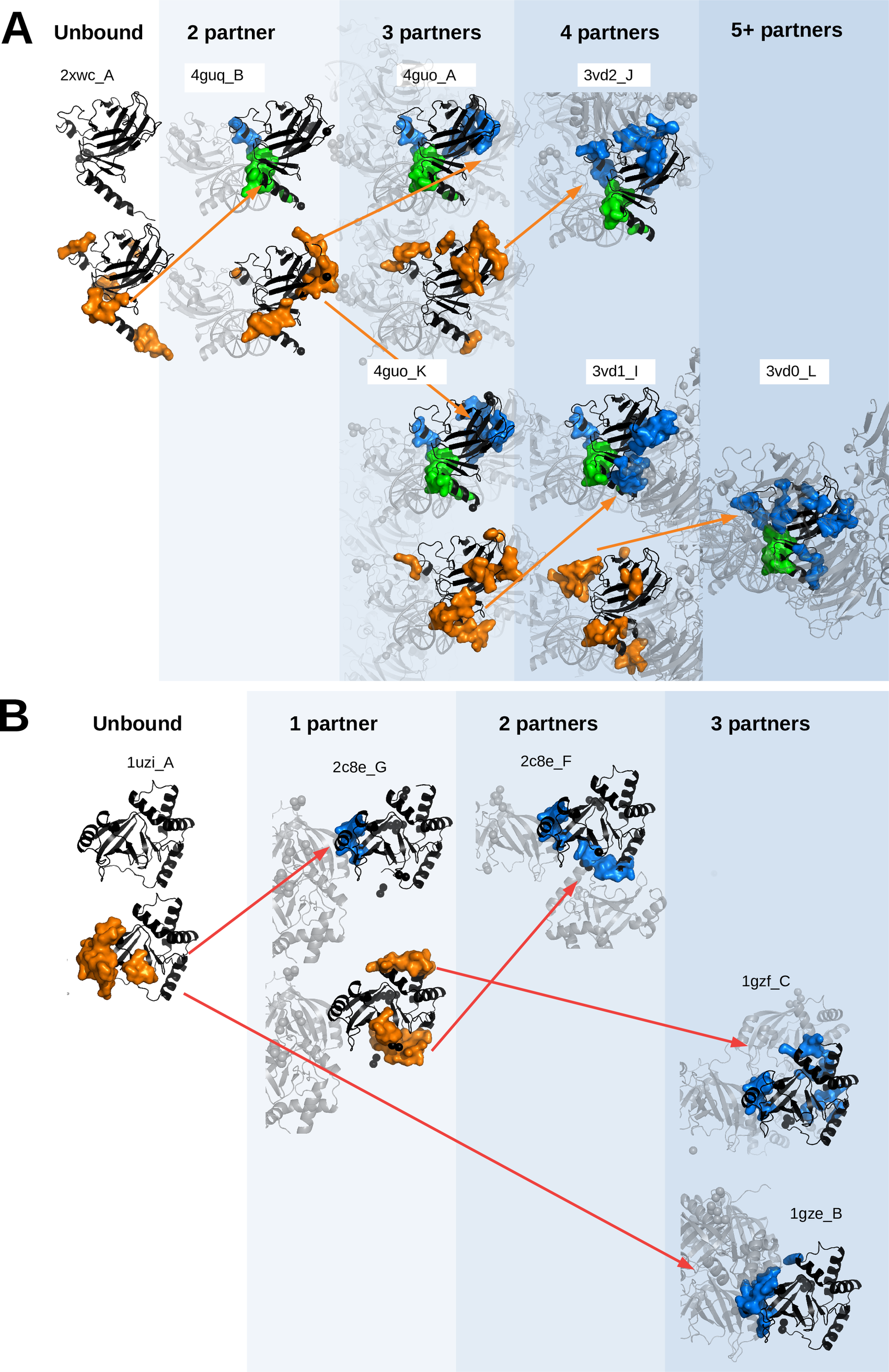

Supplement: S11 Fig — A We show the change of DRs and IRs in the p73 DNA binding domain (cluster 3vd1_D) along the orange tree branch of S2(A) Fig. In B, the change in the C3 exoenzym (cluster 2c8g_A) along the red tree branch of S2(B) Fig. (TIF) [file pcbi.1008546.s012.tif]

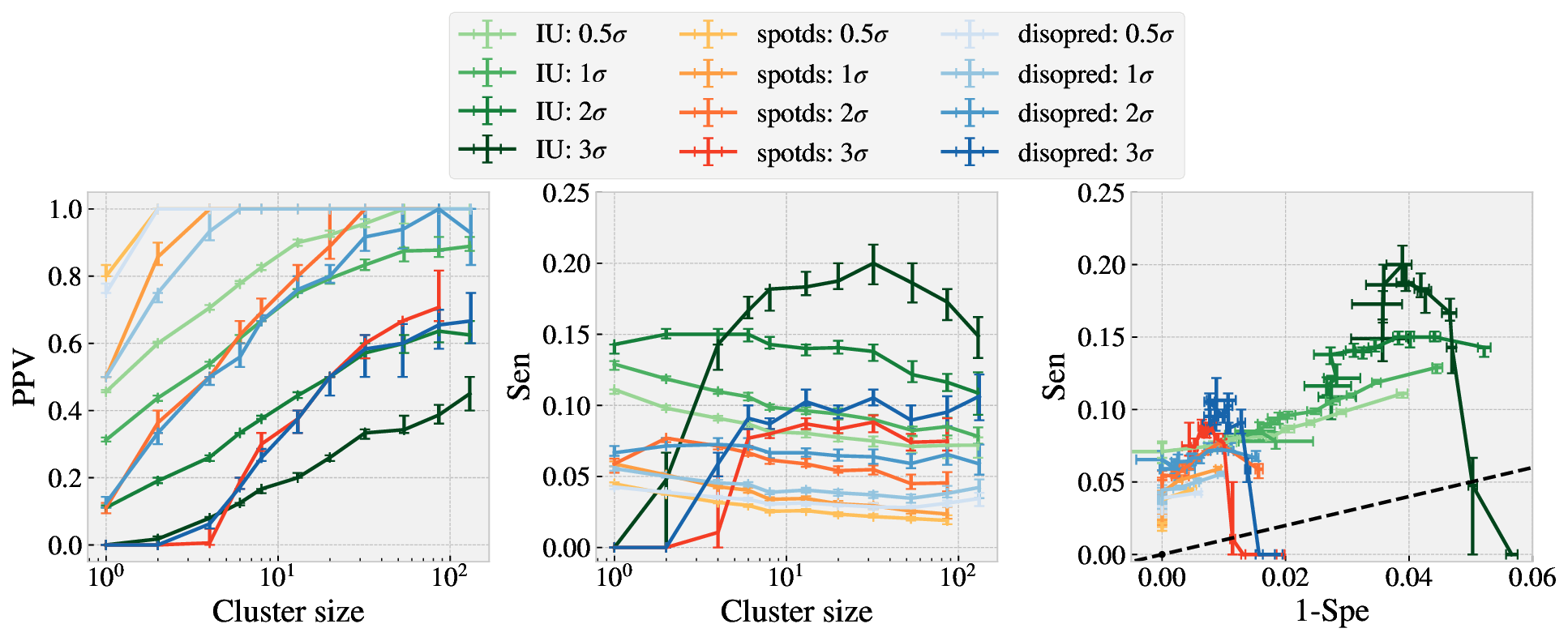

Supplement: S12 Fig — We compare the predictions from the disorder predictors discussed in the main-text, once removed the forever missing residues of the cluster, with our measures of the USDR. In A we show the PPV of the disorder predictions with respect to the different definitions of soft disorder, as function of the NDI. In B, we show instead the Sensibility. In C, we show the Sensibility versus the Specificity for the different NDI bins. (PNG) [file pcbi.1008546.s013.png]

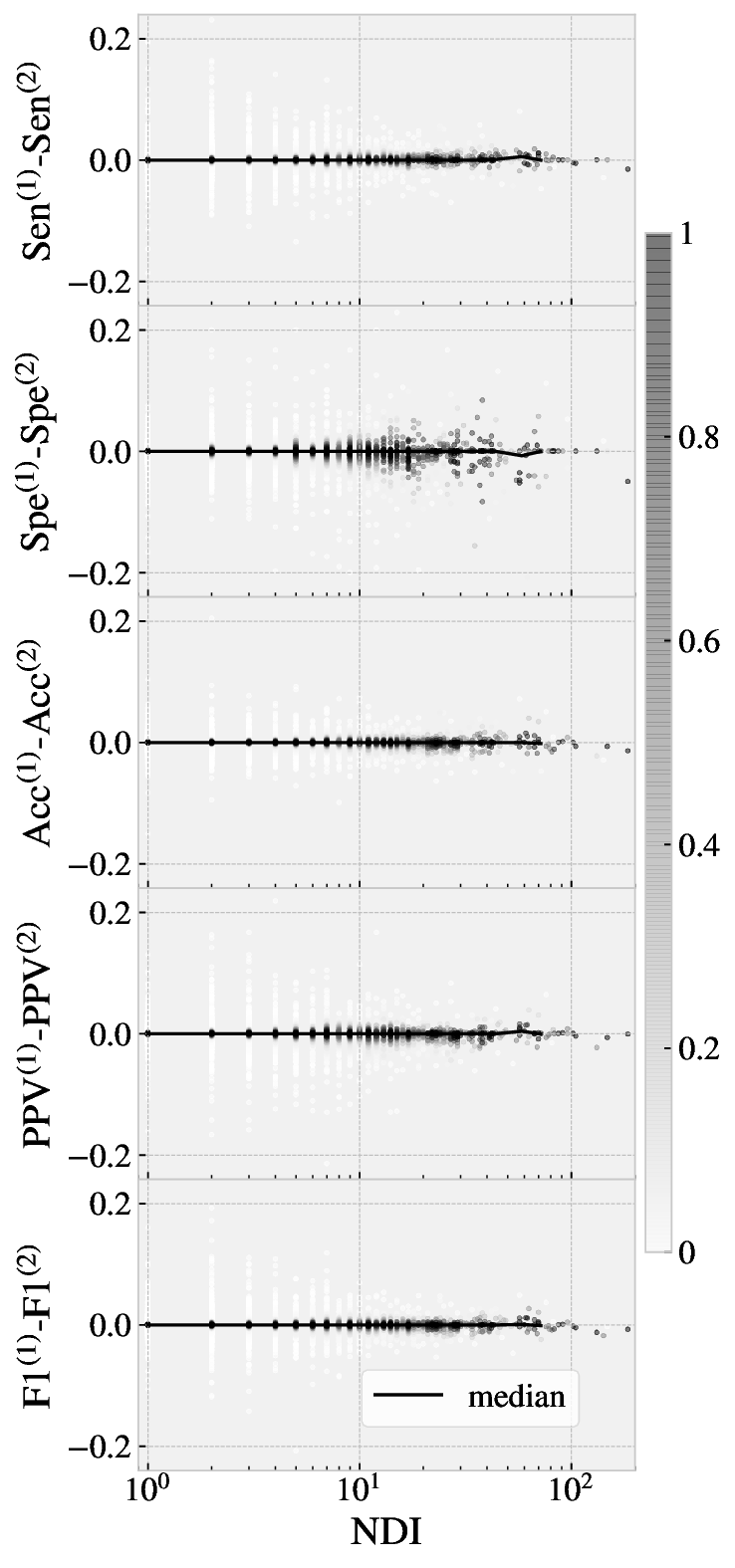

Supplement: S13 Fig — We compare the metrics quantifying the agreement of USDR and the UIR shown up to this moment, or if the representative is chosen randomly (instead of by the clustering algorithm). Each cluster is shown as a dot, and the shade of colour indicate the density of points for each bin. We see that the choice of the representative has no systematic effect in the results. (PNG) [file pcbi.1008546.s014.png]
