# Supplementary material for: The complexity of protein interactions unravelled from structural disorder
Source: PLoS Comput Biol. 2021 Jan 8;17(1):e1008546. doi: 10.1371/journal.pcbi.1008546 (PMC7846008; doi:10.1371/journal.pcbi.1008546)
Supplement: Attachment — Submitted filename: review4.pdf [file pcbi.1008546.s015.pdf]

Dear Editor,

Please find attached a mayor revision of our manuscript “The complexity of protein interactions unravelled from structural disorder”, which we are hereby submitting for a second round of reviews, we hope it is now considered for publication in PLOS Computational Biology.

First of all, we would like to thank the referees for their careful and constructive reading of the previous version of the manuscript. Indeed, their questions and concerns have allowed us to understand much better the problem tackled, and we believe that this new version of the manuscript is not only clearer but also the analysis has been improved in the content and layout.

I pass to summarise the changes introduced in the new version, more details will be given in the direct answer to each of the referees:

- We have reformulated the confusing terminologies as suggested by the referees. In particular, we have introduced a distinction between the standard *intrinsic disorder* (extracted from the missing residues in the X-ray crystallographic structure), and our definition of disorder (discussed in the manuscript as *soft disorder).* We have included a new Fig. 1 that summarises the differences. We have also avoided the term predictor all over the text since we agree with the referees that the correct term should be interface propensity.

- In order to answer the claims of Referee #2 about correlations in the analysis, we have modified the layout of our analysis to avoid mixing missing residues and high B-factor residues in the same observable. We now study in parallel both kinds of disorder and we show that they are actually related (residues that suffer a disorder-to-order transition have normally a high B-factor and so do direct neighbours of missing residues). In addition, we show that only a very small fraction of all the residues that suffer a disorder-to-order transition do it to become part of an interface. Moreover, this observation is especially used to answer against the circular argument claim raised by referee #2.

- We have used the same histogram bins of Fig. 4 in the manuscript for all the figures in the paper (in the previous version we had used several bin widths). In the new analysis, we also included some extra clusters (+1660) that had been excluded in the previous version because of an undetected error in the code.

- We have included a comparison of our analysis with the output of several disorder predictors. We show that these predictors are blind to the progressive change of structural disorder upon binding, but also that they tend to predict mostly the set of missing residues that never get structured in neither of the structures of the cluster. Yet, when wrongly predicting as disordered a structured site, this site tend to be characterised as soft disordered. Given the fact that the size of the disorder predictions are rather small in comparison with the interfaces, and that normally predict regions where interfaces cannot be found by definition (at least in the current version of the PDB), we argue that these predictors are not related to an interface propensity, unless they are used to predict the negative regions (where no interface can be placed). Even if the forever missing residues are removed from the disorder predictions, the fraction of those residues that end up in the interface is lower than the expected value for no correlation.

- We have rewritten the manuscript to improve its clarity.

Point-by-point answers are provided below and changes in the text are accordingly highlighted in red as required.

Reviewer's Responses to Questions

Comments to the Authors:
Please note here if the review is uploaded as an attachment.

Reviewer #1: This is a great study with outstanding potential. Although the manuscript is generally well-written, there are sevearl issues that need to be addressed.

We thank the referee for describing the paper as having an “*outstanding potential”* and for providing us with many useful suggestions. We have tried to address all the issues pointed by the referee, we pass to answer each of them.

1) List of all the abbreviations used in the manuscript should be provided.

We thank the referee for pointing out this issue since it had been completely missed and improves notably the legibility of the text. We have included a list at the end of the manuscript.

2) Reference 1 is wrong. As far as I know, the cited book has only one author, Dr. Peter Tompa.

The reference is now corrected.

3) Sentence on lines 20-23 is not clear and should be rephrased: "It is known that IDRs enhance the protein flexibility, and contain a large number of short linear motifs [9], which contributes increase the number of conformational states, the protein's promiscuity [10–13] and its functional versatility [14, 15]."

Sentence on lines 28-29 should be reworded for clarity "In addition, IDRs often structure after the binding, suffering a so-called disorder-to-order transition [10], ..." I think that it should be something in line with: "In addition, IDRs often become at least partially structured after the binding, undergoing a so-called binding-induced disorder-to-order transition [10], ..."

We have reformulated both sentences as:

Intrisically disordered proteins are known to enhance the protein's flexibility, and with it, increase its number of possible conformational states. Furthermore, many intrisically disordered proteins and regions possess many disordered interaction motifs that can be used to form alternative complexes and assemblies. All together, structural disorder is regarded as a mechanism to increase the protein promiscuity and enrich its functional versatility.

4) Sentence in lines 68-69 is not clear "This extended definition of disorder gathers, at the same time, the flexible and the amorphous parts of the chain". What are the amorphous parts of the chain? Should they be "ambiguous" (i.e., showing different structure in different PDB entries of similar proteins)?

We have introduced the following sentence in the text, we hope it clarifies what amorphous means:

A region having a high B-factor can indicate two distinct situations: the region is (I) floppy or flexible (either it has a mobile, not well defined structure, or it can adopt different alternative conformations), or (ii) its structure is amorphous, meaning by this that, its conformation is essentially rigid (do not change in time), but it is not reproducible in the different units that form the crystal (a.k.a. a glass compared with a crystalline solid in Solid State physics).

5) The authors considered a protein chain as bound if an interface can be measured with at least one other protein/DNA/RNA chain in the PDB complex, whereas the protein chain was classified as unbound otherwise. How about the presence in a PDB structure of small molecules and not the other protein/DNA/RNA chains? In my view, protein-based complexes should include both types of interactions – binding of other protein/DNA/RNA chains and binding of small molecules. It is well-known that small molecule binding can dramatically affect protein structure. Therefore, it would be very important to conduct such an analysis and compare the results of the analysis of these two different types of protein complexes. However, I recognize that this represents an entirely new project that probably should be a subject of subsequent study. On the other hand, I also think that a brief discussion of this issue should be added to the manuscript.

We thank the referee for raising this point. Interactions with small molecules should have an impact in the union of all the possible interfaces, and we expect these interactions to be also located at the soft disordered regions (just like the rest). However, since we did not compute the interactions with small molecules in the original design of the analysis, introducing them would have required to redo all the analysis we have done so far and it would be an enormous amount of work. The investigation of the role of interactions with small molecules will be certainly the subject of a future updates on this project. We have nevertheless added a paragraph about this in the Discussion section.

We expect small molecules binding to occur also in disordered regions, because they need to adjust easily, as proteins or DNA and RNA do. It is interesting to observe that small molecules usually bind in small conserved pockets located close to protein interfaces. The localisation of these small pockets, at the border of protein interfaces, fits with the position of disordered regions after protein binding that we could observe in our study.

6) Some aspects related to the importance of intrinsic disorder to assembly of proteinaceous machines were discussed in PMID: 24702702. Among various disorder-based mechanisms related to the assembly of protein complexes, a stepwise targeting and directional sequential assembly mechanism (binding chain reaction) was proposed there (figure 3 in that paper), where binding-induced (partial) folding of an IDP/IDR can generate a new conformation with a novel binding site for a new partner. In my view, this stepwise assembly mechanism resembles the mechanism of protein complex assembly described in the current manuscript.

We thank the referee for suggesting this paper that we had completely missed and which is completely related with our research project! It is now cited in our article and we added a paragraph speaking about

the sequential chain binding presented in the assembly of proteinaceous machines article cited by the referee.

Probably, the authors should also comment on the correlation between intrinsic disorder-based assembly and allosteric regulation.

We included a sentence and 2 new references:

Ferreon ACM, Ferreon JC, Wright PE, Deniz AA. Modulation of allostery byprotein intrinsic disorder. Nature. 2013;498(7454):390–394. doi:10.1038/nature12294.35.

Berlow RB, Dyson HJ, Wright PE. Expanding the Paradigm: IntrinsicallyDisordered Proteins and Allosteric Regulation. Journal of Molecular Biology.2018;430(16):2309–2320. doi:10.1016/j.jmb.2018.04.003.

in the Discussion Section.

There are several linguistic and stylistic issues in the current version of the manuscript. Therefore, the manuscript should be carefully edited by native English speaker or by a professional editor.

We revised the language throughout the manuscript.

Reviewer #2: The manuscript by Seoane & Carbone describes the correlation between intrinsic disorder and interaction interfaces as calculated on a clustered version of the Protein Data Bank (PDB) from June 2019. In its present form, the paper appears problematic for various reasons:

Referee #2 is very critical of our work. Even if we do not agree with most of the criticisms raised, the exercise of proving them wrong has allowed us to understand many features that we had not understood before. Motivated by this new insight, we have modified slightly the analysis layout, and we think the new version is much clearer and illustrative. We wanted to thank the referee for that.

1. Terminology. The authors seem to use non-standard, somewhat cereless or confusing terminology, e.g. referring to "intrinsic disorder" (which they largely use themselves) as "structural disorder" in the title and some other places. Likewise, when clustering their PDB proteins they refer to "homologous families of proteins" to groups with > 90% identity and > 90% coverage (not "length") which are essentially the same protein. Unfortunately, a number of these issues detract considerably from the clarity and understandability of the manuscript.

We thank the referee for pointing out which terminologies we use are not standard in the literature. The new changes should allow the readability of the article by the community with no difficulty. In this direction, we also introduced a new figure (Fig 1), to clarify the notion of disorder we use.

On the term structural disorder: In the present version of the manuscript, we have tried to separate systematically the term intrinsic disorder (associated to the lack of structure in the X-ray experiments) from our definition of disorder, that we call *soft disorder*. We continue using the term of structural disorder as a more general term (that includes both definitions) containing different degrees of disorder in the protein structure (thus including also flexible or floppy regions that appear stable in the structure, not only the missing residues). Furthermore, in the analysis, we now use the term intrinsic disorder regions (IDR) exclusively for the protein regions that are missing in all the experiments available, and the term disorder-to-order regions (DtO) for the residues that are sometimes missing and sometimes structured in the clusters of structures.

For protein homology at >90% seq id, we explicitly refer to them as being “highly similar proteins” (pp2 line 51) or "nearly identical proteins" (pp4 line 106), and we have completely removed the term homologous families in the text. We now simply speak of clusters of structures containing a particular protein.

2. The authors pack together missing PDB residues and residues with B-factor > 1 standard deviation above average for the chain as "disordered". This reviewer has to take issue as there is no statistical analysis on the relative abundance of the latter vs. the former.

In the new version of the paper, the missing residues and high B-factor residues are studied separately. Yet, the relative size of both kinds of reasons is accessible in Fig. 6A, in particular for the clusters containing just one structure.

In particular, this construction is tautological when applied to "predict" interactions (see next point). Moreover, it cannot be said that residues with a B-factor of, say, 30 when the average is 20 and SD 5 can be considered "disordered" in a structure. Quite far from it.

We have replaced the term predictor for the term interface propensity, which is more accurate to the concept we had in mind. Yet, we don't agree with the statement of tautological construction, we will argue against this below.

The referee also states that the residues with high B-factor are very far from being "disordered". We consider the referee is wrong, and to justify this, we have included a new figure (Fig. 6 in the new manuscript). Of course, residues must be structured to have a high B-factor (which means that they cannot be missing residues), but yet their structure is not well determined (this can occur because they are flexible parts of the protein, because the protein oscillates between alternative conformations or because these region get frozen in amorphous-like configurations which are not reproducible in the different units of the protein crystal). All this situations imply a certain degree of structural disorder (even if it is not the sense of the standard intrinsic disorder). Beyond this, we show there is also some overlap between the intrinsic and soft disorder: the residues that suffer a disorder-to-order transitions have a high B-factor when structured, as we show in Figs. 6--C and 6—D. We also show in Fig. S3--C that residues adjacent to missing regions also tend to have high B-factor.

3. Circular argument for missing residues. The authors look at large clusters, where many PDB structures correspond to the same (or very similar) protein solved with different binding partners. They use this set to establish residues which are missing in structure A, but solved in structure B. Knowing how crystallography works, it is trivial to say that the same protein alone would be crystalized in the same way (i.e. same missing residues) over and over again. In many cases the crystallographer would even eliminate possibly disturbing parts from the construct to help crystallization. However, the author's method only picks up these residues as "disordered" mainly because there is a different strcuture where the coordinates are present. Hence, the new structure "proves" both "disorder" and "interaction"... neither of which exists in absence of the other.

The referee claims a circular argument for the missing residues because they are only included in the analysis when they get structured, and he/she argues that this normally happen precisely to form an interface. This last argument is false : most of the times a missing residue gets structured, it is not located in the interface (we show the data in Fig. 6—B). Actually, end up in the interface is quite rare, it only happens, in average, a bit less than in a 20% of the structures in the cluster and in less of a 5% if we consider the median! This means that, most of the times we label a residue as disorder-to-order(DtO) it is not part of the interface (at least at that precise structure). The referee might still argue that the small fraction of DtO residues that almost always end up in the interface is enough to generate our correlation between DtO or soft disorder regions and interfaces.However, it is not. Indeed, if we do the same analysis (that is removing the always missing residues) from the predictions obtained using intrinsic disorder bioinformatics' predictors (see Fig. 13—D), absolutely no correlation with the interfaces is observed. Neither should we get any correlation from the disorder in unbound structures if the referee's argument were valid. An additional ingredient is necessary, and we see that it is much better captured by the B-factor than by the missing residues.

Nevertheless, in order to avoid this criticism concerning the missing residues, we decided to reproduce the same analysis exclusively with the high B-factor residues in the new version of the paper. In the new analysis, no missing residue is removed from the sequence in the metrics, unless we discuss the disorder-to-order residues as a group.

4. Flawed argument for "high B-factor" residues. The point above becomes worse for high B-factor residues. In this case, structure B will have lower B-factors compared to A precisely because there is a binding partner increasing rigidity. At this point the correlation becomes meaningless.

At this point, we think that the referee misunderstood our union procedure. We have tried to explain it better in the new version of the manuscript.

We never consider differential information by comparing two structures A and B like the referee is claiming. When a residue is missing in some structure, we consider it. When a residue has a high B-factor in some structure, we consider it. Our knowledge accumulates on the independent analysis of structures for a given protein. This means that we do not check whether the new structure has a lower B-factor that another one, not even if the B-factor fluctuates or it doesn't among the cluster structures. We agree that if we did so, it would be a circular argument, because one would need an interface to see a decrease of the B-factor. But we do not do that. We just know if a particular residue has been tagged at least in one of the PDB structures of the cluster as having high B-factor, and this is what we use for the analysis. This would be a trivial correlation if interfaces had normally a high B-factor, but it is actually the opposite (even if the effect is not strong, see Fig. 6—D. In general we measure a high B-factor in a structure without the interface, and later we find structures with interfaces in that precise region, but not on the

other way around. Another proof that this correlation is not “meaningless” (as described by the referee), is that we observe also correlation between the union of soft disorder measured only in the unbound structures of the cluster, and the union of interfaces measured in the bound structures of the cluster (see new Fig. 10). A situation where both observables are obtained using completely different structures.

5. Key observation as "predictor". Even assuming that there is a value to the observation, which this reviewer does not believe, it is not possible to sell this as a "predictor". Without multiple structures, the method will not be able to "predict". With multiple structures, the "prediction" is trivial and the performance metrics meaningless. Again, this may be due to a careless terminology. The authors should write about analysis rather than prediction.

As we already discussed above, we agree with the referee that the term ‘predictor’ is careless terminology. It does not appear any longer in the new version. Yet, we do find important to keep discussing the metrics, not as a prediction, but as a measure of how well the union of soft disordered residues covers the union of all interface regions. Which was our goal also in the previous version of the paper. Formally it is not a prediction because we accumulate all the information of all the structures in the cluster to compare both observables, we do not know which structures come first. However, there are two situations in which soft disorder is acting as a predictor (even if not a precise one). The first one, is when we analyse separately the unbound structures (unbound structures carry information about the bound structures) and in the examples of progressive assembly we show in the paper(that is, Figs. 11 and 12 of the main-text, and Figs.S9 in the Supplemental Material).

6. Several statements, especially in the abstract and discussion, have to be toned down in light of the points above.

Specific points:

** The text should be re-written for simplicity and clarity. Many sentences are quite winding, with qualifying statements detracting from the logic of the argument.*

We have completely rewritten the text. We hope the referee finds it clearer now.

** Clustering. The authors should try to use SIFTS and UniProt accessions, available from PDBe, to use a standard procedure.*

The goal of this study was to study the redundancy of a protein laying in different structures. For this, our current clustering method works perfectly. We agreed with the referee that the term homologous families was not accurate, so we have removed it. We will study if we still find correlation in distant homologous families in the future, and we will keep in mind the referee’s comment.

** Clarify PDB "old format".*

We meant the PDB file format. We have clarified this in the manuscript as well.

** The "INTerface builder" method should be better explained.*

We added a paragraph describing the method in the Methods Section (pp 10).

** Figures will benefit from clearer captions. E.g. in Figure 1, why is "2bio_A" in the center, yet the legend talks about "4ibs_A"?*

The name of the cluster is "4ibs_A", "2bio_A" is one of the structures of the cluster. Each cluster is named by its chain representative, which is just one of the structures of the cluster (chosen automatically for its precision or R-value). We have tried to clarify the captions.

** The order of figures does not match the text.*

We have solved the problem. Thanks for the remark.

** "Results" appears twice s a section heading.*

We removed the redundancy.

Reviewer #3: The study, while certainly interesting in design, sticks rigidly to its scope, when an obvious extension (particularly given the conclusions) would be to compare results using a few disorder predictors. The findings in the study do not tend to support the conclusions, and negative results displayed in the figures are glossed-over in the text. Overall, I believe that there are a number of issues that need to be addressed before it would be ready for publication. It would also greatly benefit from a round of grammatical revision.

We thank the referee for the constructive comments. We have tried to include all of them in the text.

Issues:

• *There are reasonable citations to support the work being done, but insufficient citation of previous similar works (e.g. PMC2646137) nor a comparison of conclusions.*

We thank the referee for the reference. We were not aware of this work, and that's why we did not discussed it. We have included several new references (including the referees' suggestion) to amend this problem.

• *At a number of points, it is mentioned that computational disorder predictors don't take into account all information, thus are relied upon to generalize. An actual comparison of the predicted disorder (from a couple of better known predictors) to disorder defined in this paper would be useful. A comparison of results between using predicted and measured disorder to define interfaces would be even better!*

We have included in the Discussion a new analysis and a whole new Figure (Fig.13) comparing our results with three predictors (IUPred, SPOT-Disorder and DISOPRED) and with predictors of missing residues that structure upon binding (ANCHOR and Disopred3). We discuss that, by construction, these predictors are blind to the change of the disorder (both soft and missing) upon progressive binding. In fact, we observe that all 3 tend to predict the missing residues that remain missing in all the structures of the cluster, predicting only a small fraction of all the disorder-to-order residues extracted in our analysis from the redundancy of the PDB. We also observe that when they wrongly predict a structured site, this site tend to be labelled as soft disorder in our analysis.

Finally, we check the correlation between being predicted disordered, and belonging to the union of all the interfaces. We find that the overlap between both regions tend to be lower than the expectation for decorrelated observables, indicating that, even if disorder predictors seem not to be suitable for highlighting where the interfaces would be accommodated, they might be helpful to tell us were they are not.

• *How is the reference sequence for each cluster determined? Do the results change much if a different (randomly selected?) member of the cluster is used as the reference sequence?*

All the sequence of a cluster are essentially equal (the are 90% similar), and the current choice of the representative was already quite random, so we did not expect any noticeable effect. Yet, we have repeated the analysis choosing randomly the representative, as argued, and no systematic effect was found. We include the results in Fig.S10.-

• *As well as 'missing' residues, any residues with a B-factor greater than 1SD from the chain mean is classed as disordered. However, this means that around 1/3 of every protein is defined as disordered regardless of the quality of the crystal structure. (2/3 of the data should lie within 1SD of the mean). What would be the result if a static threshold were used instead?*

Actually, one should expect a 1/6, not 1/3 (because we only use the values ABOVE the mean+SD, that is, the right side of the tail). The identification of a good static threshold is tricky, because the B-factor changes with the resolution. Furthermore, the B-factor of the different disordered regions of interest (interfaces, disorder-to-order residues, disorder predictions…) is not well separated when one considers the unnormalized B-factor (see new Fig.S3). We believe that it should be possible to use a static threshold but a lot of fine-tuning should be done. Yet, we have studied other possible thresholds for the definition of the normalised B-factor (as we show in Figs. 6, 8 and 9.

• *"We consider two interfaces as different if the number of non-mutual AAs in the interfaces is larger than 5% of the sequence length. This means that two interfaces that are concentric or slightly displaced, but one significantly larger than the other, are counted as different interfaces." Does this approach introduce a size bias due to surface-to-volume ratio? Is 5% not too much? I consider a 100 AA protein where 15 residues are interface. This says 5 residues can be different (1/3 of the interface) and it is still considered the same interface. What happens when this threshold is lowered?*

We decided to keep the threshold of 5% in the main text to avoid changing the figures. We show results as function of the NDI computed with a threshold of 1%, or just the size of the cluster in Fig.S6. Curves are less noisy using the 5%, but these other selections lead to qualitatively similar results.

• *"lead different curves, as shown in Fig. S3." This actually refers to Figure S2B. Also, while the full randomisation gave a distinctly different curve (more connected regions at lower NDI) the site randomisation was actually very similar to observed DR curve.*

We have checked the references to the Supplemental Material in the new version. We agree that the site randomisation describes a more similar curve, but it still gives lower values (incompatible with the errors) than when using the experimental “disorder”. We have nevertheless added a sentence in the main-text differentiating both situations.

• *So, to clarify: your predictor is literally: if it is disordered in the UDR: it is predicted to be an interface in the UIR? The text describes the DR being used to predict the IR, which isn't possible since it was stated earlier that they rarely occur in the same place. I think you mean UDR and UIR.*

We have removed the references to the predictor in the text. But the referee is correct, we meant the UDR and UIR.

• *Is an estimator the right term for a prediction quality score? It is at best, misleading: implying that these are estimates of interface propensity rather than quality of interface predictions.*

We strongly agreed with the suggestion of the referee, and we speak of estimates of interface propensity in the text (instead of predictor). We still kept the estimators because we wanted to quantify how good the match between the USDR and the UIR is.

• *The Bio.pairwise2.align.globalxx() function has no gap penalties. While this is much less of an issue with limits on sequence identity and length, it can still insert gaps into the reference sequence. How is it handled when a reference sequence gap is labelled as DR/IR?*

If a gap is labelled as DR or IR, then this information is not included in the analysis. Nevertheless, we do not think this is of much importance here, because all the sequences are almost identical.

• *Figure 1. An interesting and well-constructed figure, however the text overlaying the radial lines hinders reading it (the radial lines can probably be removed, and simply state that some complexes were excluded).*

We followed the referee’s suggestion and removed the lines.

• *The x-axis of Figure 3A should be 'number' rather than 'size'*

Changed, thanks.

• *With all the logarithmic bar charts / box plots it would be nice to have some graphical indication of how bin size changes on the logarithmic axis.*

We added a subplot in the new Fig.5 with this data.

• *Figure 5. Performance of this predictor looks considerably worse than is described in the text. It is essentially worse-than random guessing for predicting interfaces when the NDI is <10 (which covers the majority of clusters). Instead of presenting these 5 different metrics, a ROC curve, or a PR curve would give a better idea of method performance.*

Even if we do not discuss it as a predictor, we have replaced the 5 different metrics with a ROC curve (see Figs. 9 and 10), and discuss the goodness of the match in terms of a threshold in the number of interfaces.

• *Figure 8A. While the disordered regions do bind, these are also some disordered regions which never bind (the lower loops of the 5clx complex). Is there an explanation for these?*

This is hard to discuss, because we do not know all the possible interactions of a given protein. Yet, we do not claim that this association between soft disorder and binding sites is perfect. High b-factor includes many possible dynamic situations, and it is possible that not all of them help accommodating interfaces. We still need to study this.

• *Figure 6 C and D are never referred to in the text. I believe that references in the text to figure 6B actually refer to figure 6D, while B and C should be part of A.*

Thanks for pointing out the error.

• *The discussion is very vague and simply re-iterates the abstract. It touches once more on computational disorder prediction methods and how they can be improved greatly, however never*

compares results from them. Given that pathological implications were touched on in the introduction, this should also be expanded upon here. Just further discussion about the implication of the results in general.

We have now included an extensive discussion on this matter.

Have all data underlying the figures and results presented in the manuscript been provided? Large-scale datasets should be made available via a public repository as described in the *PLOS Computational Biology* data availability policy, and numerical data that underlies graphs or summary statistics should be provided in spreadsheet form as supporting information.

Reviewer #1: Yes

Reviewer #2: Yes

Reviewer #3: None

PLOS authors have the option to publish the peer review history of their article (what does this mean?). If published, this will include your full peer review and any attached files.

If you choose "no", your identity will remain anonymous but your review may still be made public.

Do you want your identity to be public for this peer review? For information about this choice, including consent withdrawal, please see our Privacy Policy.

Reviewer #1: Yes: Vladimir N. Uversky

Reviewer #2: No

Reviewer #3: No
