# Supplementary material for: The complexity of protein interactions unravelled from structural disorder
Source: PLoS Comput Biol. 2021 Jan 8;17(1):e1008546. doi: 10.1371/journal.pcbi.1008546 (PMC7846008; doi:10.1371/journal.pcbi.1008546)
Supplement: Attachment — Submitted filename: response_reviewers_round2.pdf [file pcbi.1008546.s016.pdf]

Reviewer's Responses to Questions

Comments to the Authors:

Please note here if the review is uploaded as an attachment.

Reviewer #1: In my view, all the critiques were adequately addressed and the manuscript was revised accordingly.

We thank Vladimir Uverski (Reviewer #1) for his comments, remarks and enthusiasm about our work all along the process.

Reviewer #2: The manuscript by Seoane & Carbone has been extensively modified. Unfortunately, this reviewer has not been swayed by the authors' "exercise to prove them wrong".

We would like to thank the reviewer for his/her remarks that helped us to reconsider our statements and argue for their correctness based on extra evidence. We would like to underline that many of the changes of our second version of the manuscript have been made to answer to Reviewer #2 questions and doubts. The manuscript improved in clarity based on these changes and the results are sharper due to the new analyses.

1. X-ray crystallography. The authors seem oblivious to the problems arising from different resolutions and crystal quality. What they attribute to "disorder", i.e. the difference in missing residues and/or high B-factors, does not consider diffraction quality as a much more obvious answer.

Our definition of high B-factor is normalized in the protein chain. This means that it highlights the regions in the chain where diffraction experiments have more difficulties to resolve the structure, at any resolution. Data is further averaged over a large quantity of clusters and compared to the expected statistics for a random location of these regions.

To explicitly address the referee concern about the quality of crystal's resolution that could influence the claims, we have included a new figure reporting our previous analysis and a new one redone by computing clusters exclusively based on structures with resolution ≤ 2.5A. The new set of clusters is now smaller and each cluster tends to have a lower number of structures (and number of different interfaces). Nonetheless, we observe essentially the same results. Namely, in the figure, we report the median PPV of the USDR matching the UIR using either the old clusters (with no limit in resolution), or the new clusters where only high-resolution structures are considered. The effect on the resolution is seen within the error bars. The curves show that resolution has no noticeable effect.

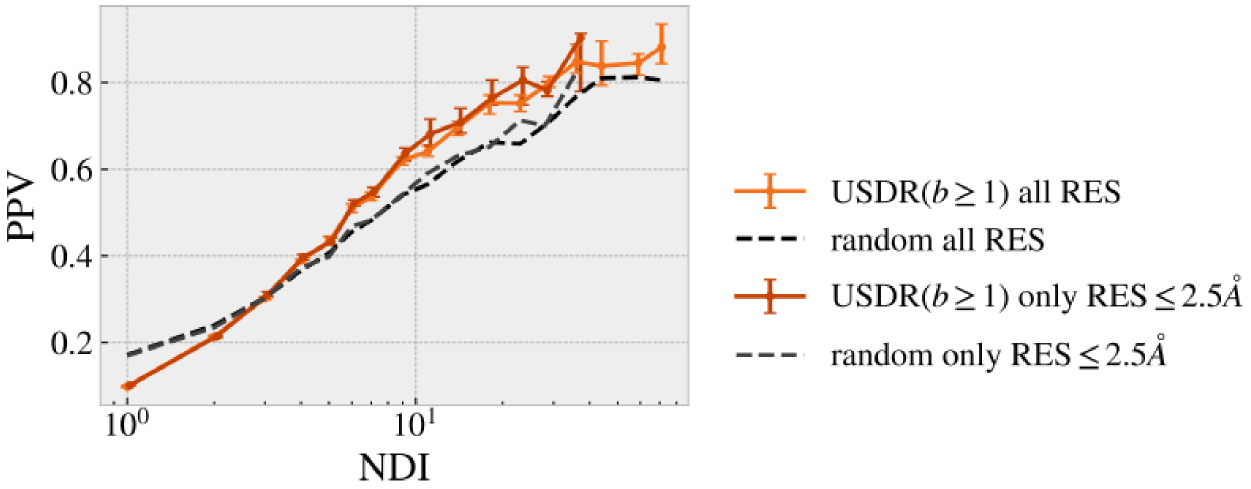

In the main text (page 4), we now mention the no noticeable effect on resolution and include this figure in the SI (Fig. S8).

2. Non-standard nomenclature and jargon. The authors have not eliminated the use of many non-standard terms. This denotes a lack of attunement to the scientific community they are trying to address. In particular, this reviewer takes issue with the term "soft disorder" in the way it has been conceived. Again, any crystallographer would probably call it just "flexible residues".

The referee seems to claim that high B-factor is just a measure of protein flexibility and that nobody in the community would understand its connection with structural disorder. In this respect, we wish to notice that reviewer #1 and reviewer #3, both members of the community, were satisfied with the definition of “soft disorder” they could read in the last version of our manuscript. This definition is now explicitly stated and unambiguous in the manuscript. In this new round, we have more extensively discussed the relationship between high B-factor and disorder in the introduction. Moreover, and most importantly, there were two important observations that we wanted to make to clarify further the idea of protein flexibility versus “soft disorder”.

Firstly, the connection between structural disorder and high B-factor is well-established in the literature, it has been studied systematically in well-known papers:

- Radivojac, P., Obradovic, Z., Smith, D. K., Zhu, G., Vucetic, S., Brown, C. J., ... & Dunker, A. K. (2004). Protein flexibility and intrinsic disorder. *Protein Science*, *13*(1), 71-80.

- Linding, R., Jensen, L. J., Diella, F., Bork, P., Gibson, T. J., & Russell, R. B. (2003). Protein disorder prediction: implications for structural proteomics. *Structure*, *11*(11), 1453-1459,

showing that there is, for instance, a large correlation between high B-factor regions and REMARK 465 regions, a fact exploited by the DISEMBL disorder predictor. Furthermore, protein disorder databases, such as MobiDB3.0,

-Piovesan, D., Tabaro, F., Paladin, L., Necci, M., Mičetić, I., Camilloni, C., ... & Parisi, G. (2018). MobiDB 3.0: more annotations for intrinsic disorder, conformational diversity and interactions in proteins. *Nucleic acids research*, *46*(D1), D471-D476.

include annotations about high B-factor regions as indirect evidence of structural disorder.

Secondly, high B-factor is not equivalent to high flexibility (even though it is commonly assumed in the literature). The reason is in the very core of the refinement necessary to extract a structure from a X-ray crystallography experiment. We just copy two paragraphs below from the book *Crystallography made crystal clear: a guide for users of macromolecular models* by Gale Rhodes (2010). Elsevier.

"There are also two important physical (as opposed to statistical) reasons for uncertainty in atom positions: thermal motion and disorder. Thermal motion refers to vibration of an atom about its rest position. Disorder refers to atoms or groups of atoms that do not occupy the same position in every unit cell, in every asymmetric unit, or in every molecule within an asymmetric unit. The temperature factor Bj obtained during refinement reflects both the thermal motion and the disorder of atom j, making it difficult to sort out these two sources of uncertainty."

"In crystallographic models, higher B-factors in sections of a well-refined model can mean that these sections are dynamically disordered in the crystal, and thus moving about faster than the time scale of the data collection. The averaged image obtained by crystallography, just like a photo of a moving object, is blurred. On the other hand, high B-factors may mean static disorder, in which specific side chains or loops take lightly different conformations in different unit cells."

A lot more discussions about the interplay of disorder, flexibility and high B-factor can be found in the recent review:

-Sun, Z., Liu, Q., Qu, G., Feng, Y., & Reetz, M. T. (2019). Utility of B-factors in protein science: interpreting rigidity, flexibility, and internal motion and engineering thermostability. *Chemical reviews*, *119*(3), 1626-1665.

To answer to the referee preoccupation that the community would not interpret correctly the notion of "soft disorder", we added and discussed the references as well as the last two points above.

3. Circular argument. This reviewer stands by its previous remarks (see points 2-4 from the previous review) and has not been convinced.

Let us first summarize all the changes we did in our previous version to answer to a possible misunderstanding of Reviewer #2 concerning a "circular argument" we could have introduced in the our reasoning:

1. We removed the concept of "prediction" from the initial version.
2. We analyze **independently** (i) the union of all the interface regions and (ii) the union of the missing and high b-factor regions (a.k.a. "soft disorder" regions) in a protein cluster and show that interface regions, on the one hand, and soft disorder regions, on the other hand, have an important (and non-trivial) overlap in the sequence.
3. We have shown that both the interface and the soft disordered regions do not often coexist in the same crystal structure, which means that "soft disorder" does not need the presence of an interface to exist (in fact, the coexistence occurs in an extremely small portion of crystals in the PDB).
4. Furthermore, all interfaces and soft disordered regions are included in the analysis regardless if the other categories are or not measured in that precise structure.

Based on these four points, there is no circularity in the reasoning. The only chink we can think of, and could have been at the origin of a misunderstanding, concerns the missing residues that get structured, which tend to be characterized as soft disorder too. The referee might be worried that we only count these residues once they get structured, and since they might get structured to form an interface, we would only measure them when they belong to the interface. In this respect, we already showed that, most of the times, when a missing residue gets structured it does not end up in the interface (the median of the fraction of DTO residues that are part of the interface is less than a 5%, see Fig. 6B in the manuscript). Since the referee might not have judged this observation to be enough to dismiss the argument, we have included a new figure. This figure is the analog of Fig 8A realized for soft disorder, where we exclude systematically from the analysis all the residues that are missing at least in one structure of the cluster. In other words, we use only the residues that are always structured. We show that there is no qualitative change compared to Fig.8A, thus proving that the signal is not a trivial correlation associated to the disorder-to-order transition. However, we stress that missing residues are correlated to the interfaces, since we showed in Fig. 8B that the DtO alone provided essentially the same behavior in PPV the soft disorder. Yet, being the DtO regions much smaller, they tend to cover only a small part of the interface, which means that the information of both kinds of disorder must be taken into account when trying to describe the union of all the alternative interfaces with other partners. These new results suggest that DtO regions behave very much like other high B-factor regions.

This new analysis is explained in the new Fig. S10 and discussed in a new parragraph in Page 7.

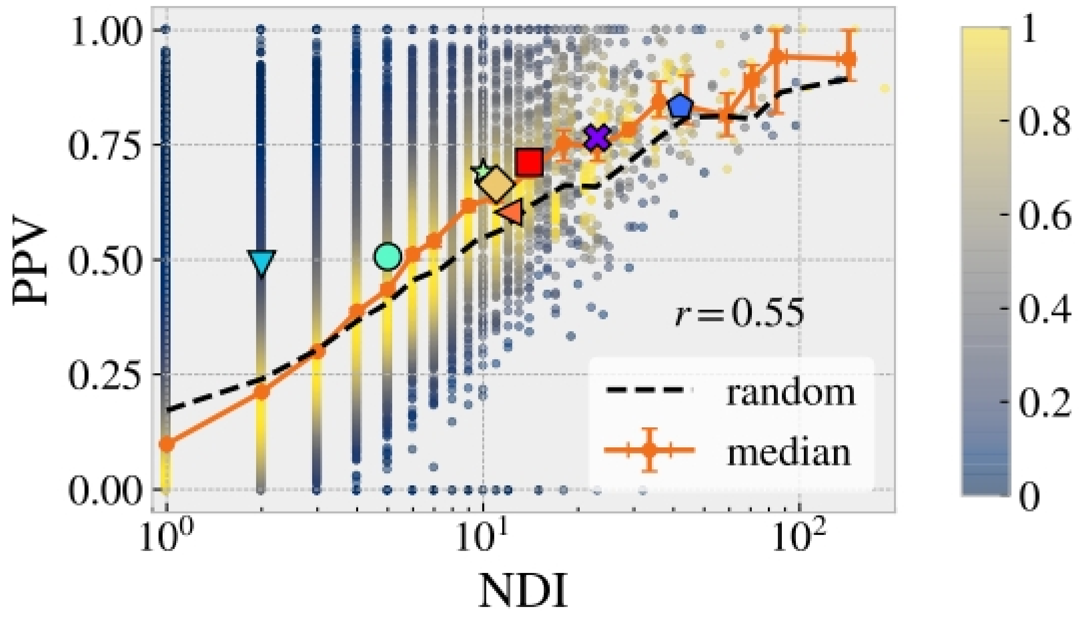

4. High B-factors. As also pointed out by reviewer #3, this definition for "soft disorder" is flawed and inflates the perceived fraction of "disorder" for perfectly rigid structures.

In order to answer to Referee #3, we considered different thresholds for the definition of soft disorder, from very high b-factor (normalized b-factor>3) to relatively low (normalized b-factor>0.5). Large thresholds tend to identify as soft disordered a large portion of the amino acids, and low thresholds a small one. Fig. 6A shows the growth of the relative sizes of the regions with respect to the size of the clusters, for different thresholds. When we compare the PPV as a function

of the NDI, we find that the USDR at b-factor>3 has a higher probability of being part of the interface than USDRs at smaller b-factors (see Fig. 8B), but covering a much smaller size than the union of the interfaces.

Also, we see that if we change the definition of soft disorder by including more rigid residues in this definition (that is, considering lower thresholds for the b-factor), the total correspondence between soft disorder regions and interface regions gets better. Of course, this definition of soft disorder includes residues that are perfectly structured (that is, not missing), but they are still more dynamically or structurally disordered that other rigid residues of the chain. As stressed above, our definition of soft disorder should also be able to characterize disordered residues that are totally rigid but which are amorphous in structure, that is, for which the position of the atoms is not reproducible along the crystal in the different unit cells.

5. Flexibility. In general, the authors seem very convinced that they have found an important concept and try to promote it in several ways which do not appear consistent with the existing literature. While "disorder" is a trendy topic, not everything is best explained with this concept. The decades old notion of flexibility is much better suited to several key observations of this manuscript.

In our paper we considered both the traditional notion of disorder (the missing residues defined through the REMARK 465 in the PDB file) and our definition of ‘soft disorder’ based on the high b-factor. We show that the residues tagged by the first definition, are also tagged as soft disordered when they suffer disorder-to-order transitions, and that both definitions provide interesting insights on protein interactions. The whole point of our study is to show that when we think about **interactions**, then the two concepts of “thermal motion” and “disorder” should be put together. It is a very **simple** observation that turns out to be powerful to capture the **independent** notion of interaction. We agree that the paper did not refer properly to the literature on flexibility and we have now included some references and comments in the paper. As clearly stated in the article from the beginning, we wish to point out that this work provides **a statistical analysis at a large scale**, involving all structures in the PDB, an analysis that was missing in the literature until now.

Specific points:

* Fig. 1. This reviewer did not find it very useful to explain the concepts as it does not address the definitions themselves in a graphical way.

We find that Fig1A and Fig1B highlight clearly that residues in a protein are labeled differently by the notion of intrinsic disorder (missing+disorder-to-order) and the one of soft disorder (missing+high-b-factor). In particular, it shows that residues labeled as “soft disorder” include those labeled “intrinsically disordered”.

Moreover, Fig 1CD highlights the dependency of precise measures from the available structures in the PDB and that the two notions as “disorder-to-order” for intrinsic disorder and “high-B-factor” for soft disorder are dependent on the available structures.

We believe that these concepts need to be well explained and that **our figure disentangles them,** helping the reading of the text and the correct interpretation of the new definition of “soft disorder”.

We have extended the discussion of this figure in the new version of the manuscript.

* The reference for DisProt is outdated.

We updated the reference. Thank you.

* Clustering. The authors have brushed away the request to use SIFTS and UniProt accessions because their method "works perfectly". The request weas not for a "better" procedure but for a "reproducible" one.

The extraction of the citation “works perfectly” from the context of our report is unfair. We believe that there is a basic misunderstanding that we would like to explain. Originally, we had understood that the referee’s concern about our clustering method referred to the definition of homologous families, which, we agreed, was based on a careless use of the term. It was not what we had in mind: we wanted to analyze crystals containing essentially the “same” protein, that is, “redundant” experiments. The referee now clarifies that he/she was worried about the “reproducibility” of the results. We have two comments to make related to this statement.

Firstly, sequences in our clusters are nearly identical: they have a 90% of sequence identity **and** the same length up to a 90%. The very high similarity of the sequences in a cluster makes no problem of reproducibility for the testing of these two conditions. We agree that this could make an issue if we were speaking of much lower sequence identity or a lack of restriction in the sequence length.

Secondly, we use exactly the same clustering method employed by the Protein Data Bank to measure its redundancy (see http://www.rcsb.org/pdb/statistics/clusterStatistics.do). Now we mention this explicitly in the manuscript. The only difference between our clusters and the lists/clusters of non-redundant sequences provided at the PDB website (for a 90% of sequence identity) is that our clusters are smaller because they satisfy the more restrictive and additional condition on the total sequence length. This point is made clear now in the Methods.

In order to avoid the problem of reproducibility, we provide, with this new version of the paper, the scripts used to generate the clusters. They are available at http://www.lcqb.upmc.fr/disorder-interfaces/.

Reviewer #3: I am happy that the authors have adequately addressed my comments.

We thank Reviewer #3 for his/her comments, remarks and support all along the process.

Have all data underlying the figures and results presented in the manuscript been provided?
Large-scale datasets should be made available via a public repository as described in the *PLOS Computational Biology* data availability policy, and numerical data that underlies graphs or summary statistics should be provided in spreadsheet form as supporting information.

Reviewer #1: Yes

Reviewer #2: Yes

Reviewer #3: None

PLOS authors have the option to publish the peer review history of their article (what does this mean?). If published, this will include your full peer review and any attached files.

If you choose “no”, your identity will remain anonymous but your review may still be made public.

Do you want your identity to be public for this peer review? For information about this choice, including consent withdrawal, please see our Privacy Policy.

Reviewer #1: **Yes:** Vladimir N. Uversky

Reviewer #2: No

Reviewer #3: No
